# Tau Protein Binding Modes in Alzheimer’s Disease for Cationic Luminescent Ligands

**DOI:** 10.1021/acs.jpcb.1c06019

**Published:** 2021-10-13

**Authors:** Yogesh Todarwal, Camilla Gustafsson, Nghia Nguyen Thi Minh, Ingrid Ertzgaard, Therése Klingstedt, Bernardino Ghetti, Ruben Vidal, Carolin König, Mikael Lindgren, K. Peter R. Nilsson, Mathieu Linares, Patrick Norman

**Affiliations:** † Department of Theoretical Chemistry and Biology, School of Engineering Sciences in Chemistry, Biotechnology and Health, KTH Royal Institute of Technology, SE-106 91 Stockholm, Sweden; ‡ Leibniz University Hannover, Institute of Physical Chemistry and Electrochemistry, Callinstr. 3A, 30167 Hannover, Germany; § Department of Physics, Norwegian University of Science and Technology, 7491 Trondheim, Norway; ∥ Department of Physics, Chemistry and Biology, Linköping University, SE 581 83 Linköping, Sweden; ⊥ Department of Pathology and Laboratory Medicine, Indiana University School of Medicine, Indianapolis, Indiana 46202, United States; # Department of Physics, Chemistry and Biology, Linköping University, SE-581 83 Linköping, Sweden; @ Laboratory of Organic Electronics, ITN, Linköping University, SE-581 83 Linköping, Sweden; ∇ Scientific Visualization Group, ITN, Linköping University, SE-581 83 Linköping, Sweden

## Abstract

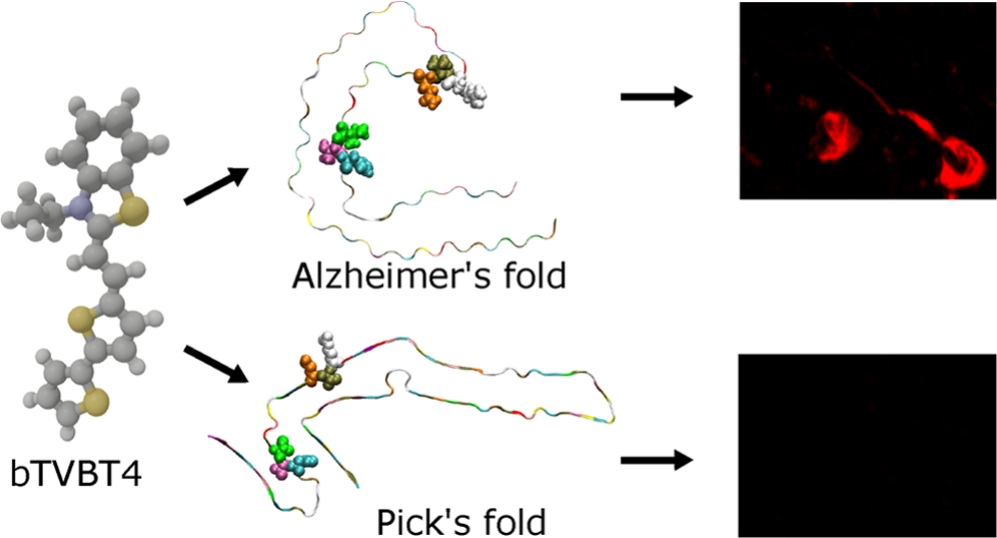

The bi-thiophene-vinylene-benzothiazole (bTVBT4) ligand developed for Alzheimer’s disease (AD)-specific detection of amyloid tau has been studied by a combination of several theoretical methods and experimental spectroscopies. With reference to the cryo-EM tau structure of the tau protofilament (Nature2017, 547, 18528678775), a periodic model system of the fibril was created, and the interactions between this fibril and bTVBT4 were studied with nonbiased molecular dynamics simulations. Several binding sites and binding modes were identified and analyzed, and the results for the most prevailing fibril site and ligand modes are presented. A key validation of the simulation work is provided by the favorable comparison of the theoretical and experimental absorption spectra of bTVBT4 in solution and bound to the protein. It is conclusively shown that the ligand–protein binding occurs at the hydrophobic pocket defined by the residues Ile360, Thr361, and His362. This binding site is not accessible in the Pick’s disease (PiD) fold, and fluorescence imaging of bTVBT4-stained brain tissue samples from patients diagnosed with AD and PiD provides strong support for the proposed tau binding site.

## Introduction

Several intrinsically disordered proteins are known to self-assemble into β-sheet filament structures (cross-β), or amyloid fibrils, associated with neurodegenerative diseases such as Alzheimer’s (AD), Parkinson’s, and Pick’s (PiD) diseases. Our current knowledge of the biogenesis and aggregation steps of amyloids has recently been comprehensively summarized, including a review of experimental as well as computational work.^1^ In AD, notably, the key proteins are amyloid β (Aβ) with 40 or 42 residues and tau with 352 to 421 residues. The domain organization of tau is complex, and disease filaments show six tau isoforms and distinct morphologies, but paired helical filaments (PHFs) in neurofibrillary tangles are central to the development of AD. These filaments are composed of a rigid and structurally ordered core and a flexible and structurally disordered coat. The molecular structure of the PHF core with protofilaments comprising residues 306–378 in tau has been determined by means of cryogenic electron microscopy (cryo-EM), revealing two C-shaped protofilaments related by helical symmetry and stacked with a rise of 4.7 Å and a twist of *ca.* 1°.^2^ Compared to AD, PiD is less common. Patients suffering from PiD have been found to have intraneuronal inclusions of hyperphosphorylated tau aggregates. However, while the symptoms of PiD may be similar to the symptoms of AD, the tau inclusion bodies associated with PiD pathology are both biochemically and histologically distinct from the aggregates of tau identified in patients with AD pathology.^3^ Tau in AD is also known to be highly phosphorylated. However, it has been shown that the phosphorylation sites predominantly reside outside the core region,^4^ and our study is consequently concerned with the pristine structure from ref (2).

**Figure 1:**
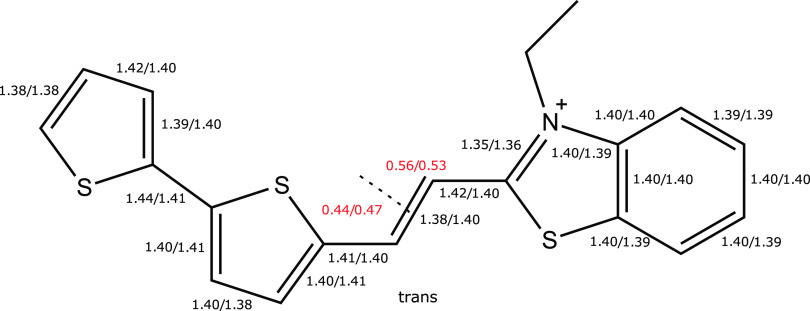
Molecular structure of the bTVBT4 ligand. Selected ground and excited state (S_0_/S_1_) bond length (Å) parameters of the π-conjugated backbone are given. Natural population analysis (NPA) charges for the bi-thiophene and benzothiazole moieties (as separated by the dashed line) in the S_0_ and S_1_ states are given in red. The S_0_ and S_1_ states are described at the levels of DFT/B3LYP and TDDFT/CAM-B3LYP, respectively.

Several methods exist to study the molecular and functional aspects of tau physiopathology.^5^ Noninvasive detection and imaging of Aβ and tau fibril deposits can be achieved by means of positron emission tomography^6−15^ and fluorescence spectroscopy. For the latter, small hydrophobic and environment-sensitive ligands have been developed most commonly as derivatives of Thioflavin T^16^ and Congo red^17^^17^ and therefore not suited for clinical studies due to their toxic character and inability to pass the blood–brain barrier (BBB). As an alternative, Nilsson and co-workers have proposed a class of ligands known as luminescent conjugated oligothiophenes (LCOs) that show aggregate-specific strong fluorescent signals upon binding to a wide range of protein aggregate morphotypes.^18^ Such LCOs have the ability to pass through the BBB^19,20^ and facilitate early-stage detection of the buildup of misfolded protein aggregates,^21−24^ and based on the bi-thiophene-vinylene (bTV) scaffold, ligands demonstrating tau-specific binding have been synthesized.^25^ On the theoretical side, we have developed a methodology to provide a microscopic understanding of the ligand–protein interactions by means of unbiased molecular dynamics (MD) and subsequent spectroscopy simulations,^26,27^ adopting results in aqueous solution as reference.^28−30^ We have applied this protocol to study the binding of the anionic p-FTAA ligand to amyloid-β and showed that the fingerprinting optical responses are associated with the planarity of the π-conjugated system.^27^ The ligand subject of the present study, bTVBT4, belongs to the bTV category and features a cationic benzothiazole (BT) moiety, see Figure 1. In contrast to p-FTAA, it demonstrates tau-specific binding, and a prime objective of the present study is to see whether or not this protein-specificity is possible to rationalize within the realm of our simple fibrillar models of the disease aggregates. In the next section, we will give an overview of the adopted methodology with guiding references to the Supporting Information (SI), where a rich amount of underlying details can be found. Thereafter follows a section discussing the key results obtained from our theoretical and experimental studies of this ligand, and we provide also here references to SI for additional information with the intent to keep the main article to the point and focused on the main message.

## Methods

The binding of bTVBT4 to the tau fibril was studied with molecular dynamics (MD) simulations, with general computational details found in SI section 1.1.4. A periodic model system representing the tau fibril was created by repeating units of tau oligomers originating from the cryo-EM structure (PDB ID: 5O3l), as described in SI sections 1.2.2 and 1.2.3. Force field parameters specific to the bTVBT4 ligand were developed with reference to quantum chemical calculations at the level of B3LYP/6-31+G(d,p), as described in SI section 1.1.2.

A detailed description of the MD simulations conducted to identify the ligand binding sites on the tau fibril is provided in SI section 1.2.4. These calculations were based on the full periodic model system for tau together with 60 ligands. To further analyze the binding modes of the main binding site, a reduced model system was created as described in SI section 1.2.5. This reduced system, consisting of 10 tau oligomer chains with position constraints imposed on the outermost chains, was also used to determine free energy profiles for the binding of bTVBT4 to the tau fibril by means of umbrella sampling. The calculations of the potentials of mean force (PMFs) for the different binding modes are described in SI section 1.2.6.

Spectrum calculations were carried out at the level of CAM-B3LYP/aug-cc-pVDZ with varying degrees of exact Hartree–Fock exchange applied in the long-range limit (100% and the standard setting of 65%). We adopted the polarizable embedding (PE) model to describe the ligand environment, and details on the spectrum convergence with respect to PE parameters are presented in SI section 1.1.7 for the case of water solution. For the spectrum calculations of the bTVBT4 ligand in the binding site, there is an additional aspect of how to properly sample all binding modes. Details on our approach to addressing this issue are provided in SI section 1.2.7.

Experimental fluorescence spectra were obtained from frozen frontal cortical brain sections from patients with either Alzheimer’s or Pick’s disease pathology, labeled with a fluorescent dye conjugated to antibodies against tau fibrils. Details on these experimental procedures are presented in SI sections 2.1–2.2. Furthermore, an experimental absorption and emission study of the bTVBT4 ligand in various solvents was performed, and details about this are provided in SI section 2.3.

## Results and Discussion

As demonstrated in a study of a flexible anionic pentameric oligothiophene (p-FTAA) targeting Aβ, binding modes of LCOs interacting with amyloids can be revealed by means of unbiased MD simulations on a time scale of a few hundred nanoseconds.^26^ It was later conclusively shown that the fingerprinting optical signal responses of this particular ligand binding are primarily due to an increased planarity in the π-conjugated system of the ligand.^27,29^ There are good reasons, however, to believe that the underlying microscopic mechanisms are different in the present case, as the bTVBT4 ligand differs in several important ways—(i) it is cationic instead of anionic with a charge that is largely delocalized over the π-conjugated system instead of being localized to carboxylate side chains and (ii) the molecular structure is not as flexible due to the vinylene double bond. It is reasonable to assume that the relevant binding modes of the ligands targeting tau are found in the structurally ordered core, and we therefore adopted the cryo-EM structure as a starting point in an attempt to reveal the most important binding modes of bTVBT4.

### Electronic Structure of bTVBT4

We first performed a complete conformer study of bTVBT4 in the electronic ground state, S_0_, and found the *trans*-conformer shown in Figure 1 to be energetically more stable by 2.4 kJ/mol as compared with the corresponding bi-thiophene *cis*-conformer. The conformers associated with variations of the three vinylene dihedral angles were also considered but resulted in increases of the energy by 6.8–58.4 kJ/mol, indicating that these conformers are not as relevant for ligand binding.

**Figure 2:**
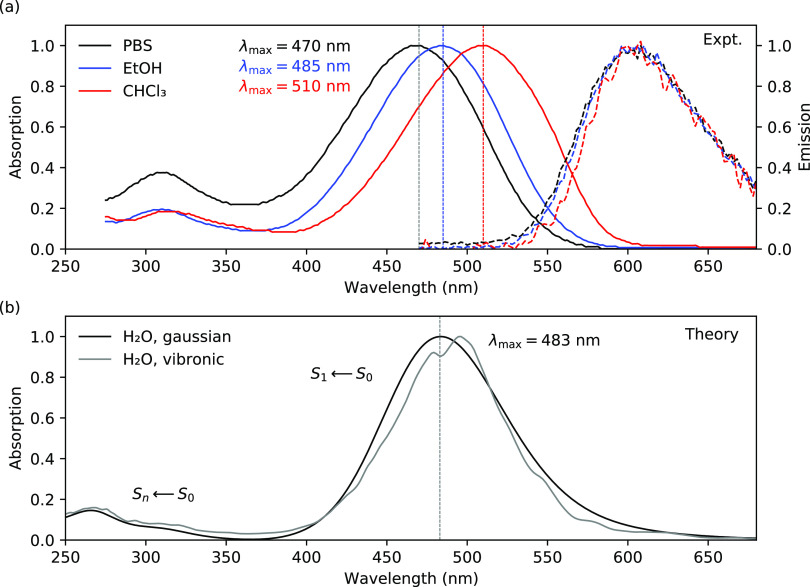
(a) Experimental absorption and emission spectra of bTVBT4 in different solvents (PBS is phosphate-buffered saline) obtained at room temperature. (b) Theoretical absorption spectra for bTVBT4 in water solution using Gaussian and vibronic line broadenings. The 10 lowest states are included in the calculations performed at the level of TDDFT/CAM-B3LYP(100%).

The positive charge of the cationic ligand is delocalized, and with a split separation made in the vinylene double bond, the bi-thiophene and benzothiazole units accumulate charges of 0.44*e* and 0.56*e*, respectively, in the ground state and 0.47*e* and 0.53*e* in the excited state, which suggests a small charge transfer associated with the S_1_ ← S_0_ transition. With a gauge-origin chosen as the center of nuclear charge, the dipole moment of the cationic ligand becomes well defined and point in the direction from the bi-thiophene toward the benzothiazole moiety. Our charge analysis predicts a lower dipole moment in the *S*_1_ state as compared to the S_0_ state, and this finding is corroborated by a quadratic response theory^31^ calculation of the (always) well-defined dipole moment difference that, here, predicts a reduction of the dipole moment from 5.5 to 2.1 D; see Figure S2 in the SI. It also agrees with the observed negative and zero solvatochromic shifts in the experimental absorption and emission spectroscopies, respectively, as presented in Figure 2a. The reasoning behind this is that, in absorption, the ground state (with its larger dipole moment and being relaxed also with respect to the slow degrees of freedom of the solvent) becomes more stabilized by the reaction field of the solvent as compared to the excited state, and hence, the transition energy increases with solvent polarity. In emission, on the other hand, the small dipole moment of the excited (initial) state causes the energy to be weakly dependent on the solvent polarity, and the ground state will not be in equilibrium with the slow degrees of the freedom of the solvent, resulting in a weak dependence also of the final state energy with respect to solvent polarity. Arguments combined explain the observed small variation of the transition energy with solvent polarity in emission spectroscopy.

An indirect probe of changes in the electronic structure between the ground and excited states is provided by the comparison of equilibrium molecular structures. For pentameric oligothiophene ligands, these changes have been shown to be localized to the three central rings and interpreted as a quite localized exciton. For these central units, the main changes amount to single–double bond inversion, resulting in a rigid planar system in the excited state.^28^ For the cationic bTVBT4, this effect is much less pronounced, and bond length parameters in the π-conjugated backbone are largely unaffected by the electronic excitation; see Figure 1. As a consequence, and as we shall see, this leads to intense 0–0 transitions in the vibronic absorption spectra.

At the Franck–Condon points, the transition state properties are determined with the use of the time-dependent density functional theory (TDDFT) method in conjunction with a range-separated hybrid exchange–correlation functional (CAM-B3LYP) to account for the charge-transfer character in the S_1_ ← S_0_ transition. There is no significant difference in the properties for the *trans*- and *cis*-conformer, as seen in Table 1. Varying the amount of exact exchange in the long-range limit from the standard setting of 65 to 100% (fully correct asymptotic behavior in charge-transfer excitations) gives rise to a small increase in the excitation energy of 0.08 eV, in line with the transition being predominantly described by a single-electron excitation from the highest occupied molecular orbital (π) to lowest unoccupied molecular orbital (π*) and where the former is somewhat more localized to the bi-thiophene group.

**Table 1:** Vertical Absorption Energies (Δ*E* in eV), Transition Wavelengths (λ in nm), and Oscillator Strengths (*f*) for the Two Lowest Conformations of bTVBT4, Where the Trans-Conformation is Shown in Figure 1^a^

	CAM-B3LYP(100%)			CAM-B3LYP(65%)		
conformer	Δ*E*	λ	*f*	Δ*E*	λ	*f*
*trans*	2.619	473.5	1.45	2.541	487.9	1.45
*cis*	2.621	473.0	1.43	2.547	486.8	1.44

a A varying percentage degree of exact exchange is used in the long-range limit (100% and the standard setting of 65%) in both cases combined with the aug-cc-pVDZ basis set.

### Spectra of bTVBT4 in Water

Experimental emission spectra of LCO ligands often display clear vibrational progressions that to a high degree of precision can be simulated with the use of anharmonic vibrational configuration interaction wave function calculations, as demonstrated in the case of oligothiophenes with an attribution of the observed vibrational progressions with a separation of some 0.18–0.19 eV to inter-ring carbon–carbon stretching and ring breathing motions.^32^ The relatively large force constants associated with ring rotations in the excited state promote such a vibrational resolution in the experiment. In the ground state, this is not the case, and therefore, in calculations of absorption spectra, it becomes necessary to also account for the slow dihedral motions that are not well described by rectilinear coordinates.

As a remedy, we propose to, in a first step, perform an anharmonic vibrational calculation on the ligand in isolation in the low-temperature limit, such that small oscillations from a well-defined molecular structure minimum provide an accurate description of the nuclear motions. For bTVBT4, this results in the identification of three vibrational stretching and angle-bending modes in the region of 1300–1600 cm^–1^ to be of prime importance for the absorption spectrum profile. The 0–0 transition dominates the absorption spectrum, leading to a strongly inhomogeneous broadening; see Figure S3 in the SI. In a second step, we adopt this low-temperature spectrum profile in combination with room-temperature MD simulations to account for the slow degrees of motion by means of the technique of snapshot averaging.^28,33^ This removes the need to introduce an *ad hoc* line broadening (typically Gaussian or Lorentzian) in the simulations and instead relies on the arguably reasonable approximation that the fast and slow degrees of vibrational motions can be decoupled.

For bTVBT4, we derived the set of molecular mechanics force field parameters based on the general AMBER force field (GAFF) with force constants describing the four dihedral rotations subsequently fitted to relaxed-scan potential energy curves obtained at the level of DFT/B3LYP, see Section 1.1.2 in SI for a detailed description. Together with the standard TIP3P force field for water, we performed room-temperature MD simulations of the ligand in an aqueous solution with a chlorine ion introduced to neutralize the system. Only the bi-thiophene cis/trans-isomerization can be observed in the dynamics, and it resulted in a statistical 50/50 conformer population in solution as compared to 35/65 in vacuum. The relative stabilization of the *cis*-conformer in solution is connected with the larger *local* dipole moment in the bi-thiophene moiety as compared to that in the *trans*-conformer.

The subsequent spectrum calculations of bTVBT4 in water were performed with the employment of the PE model using the standard Ahlstrom isotropic polarizabilities and atomic charges for a polarizable shell of thickness 15 Å and the TIP3P charges for an exterior nonpolarizable shell of thickness 5 Å—the calculations are well converged with respect to shell thicknesses, see Figure S8 in the SI. We performed 200 snapshot calculations (100 from each of the *cis/trans*-conformers), and the resulting spectra are depicted in Figure 2b, both using the vibronic line broadening as described above, as well as using a conventional Gaussian line profile, with a standard deviation of 0.15 eV. The two spectra are found to be in close agreement, indicating that the number of snapshots is sufficiently large to almost fully smear out the inhomogeneity in the vibronic line profile. Taken separately, the cis- and trans-conformations yield averaged λ_max_-values of 485 and 480 nm, respectively, and taken together, the value for λ_max_ becomes equal to 483 nm. This theoretical result should be compared to the experimental value of 470 nm in PBS solution, see Figure 2a, which amounts to a discrepancy of 0.07 eV. We retrieved a representative *trans*-conformer snapshot and incorporated the first solvation shell of 14 water molecules into the quantum mechanical region, and this, in effect, changed the transition wavelength from 480 to 475 nm. Based on these results, we deem our theoretical ligand spectrum in solution to be highly accurate and suitable as a reference for the assessment of spectral changes due to protein binding.

### Binding of bTVBT4 to Tau

Based on the experimental protofilament structure,^2^ we built a series of forced periodic, right-handed helical, tau fibril systems, all with a 2_1_ screw axis symmetry but with slightly varying twist angles. During the NPT-relaxations of these systems, the forced periodicity induced stress that at times resulted in structural kinks. Our smoothest system (with least stress) was found using 185 protofilament layers, corresponding to a twist angle of 0.97°, with a rise of 4.8 Å at 300 K, which is in close agreement with the experimental cryo-EM structure.^2^

**Figure 3:**
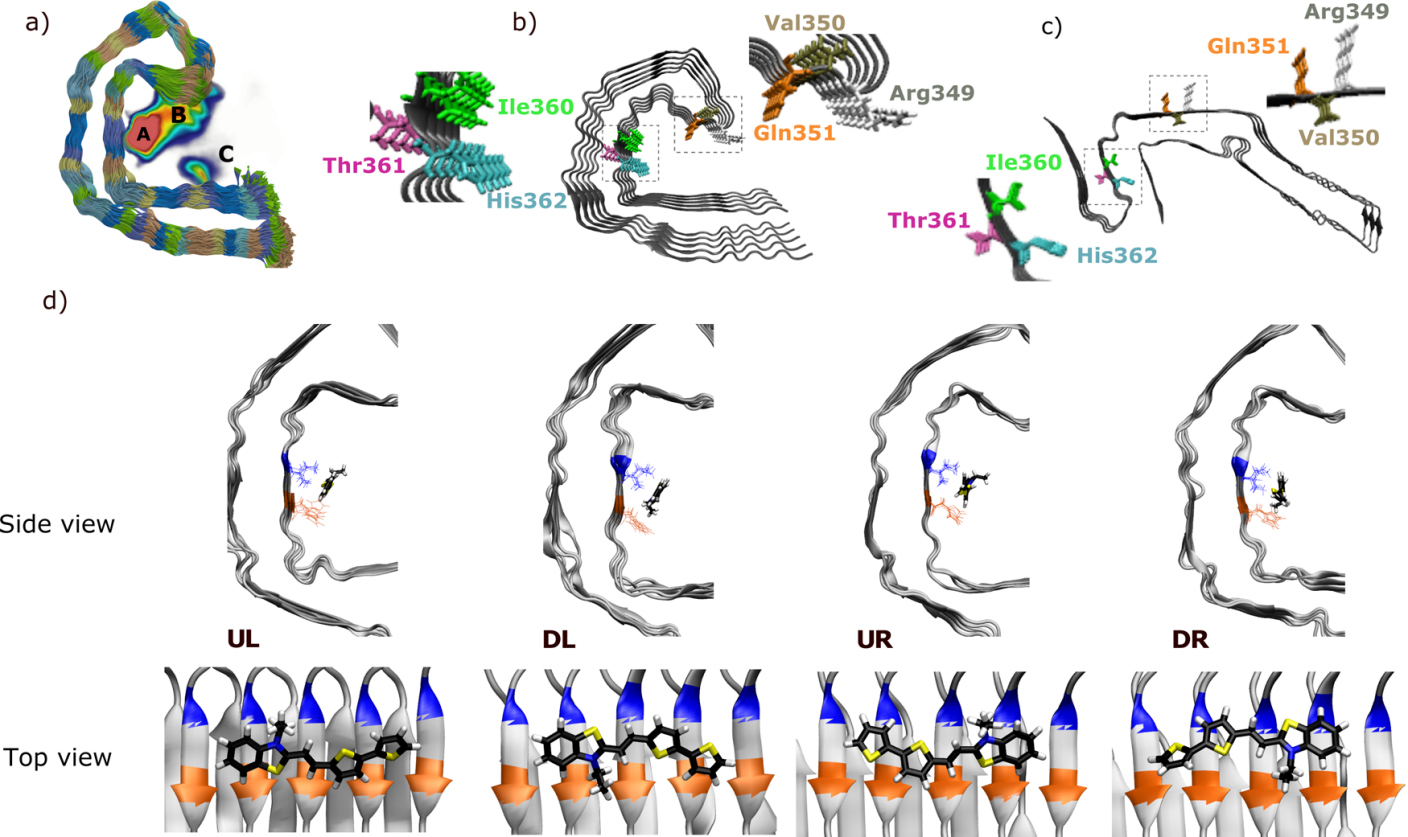
Summary of the MD simulations of the interactions between bTVBT4 and the amyloid fibrillar structure of tau. (a) Density map of the bTVBT4 interactions with the tau Alzheimer’s fold, where red indicates the highest, blue a lower, and white a zero ligand density, (b) Alzheimer’s fold (PDB ID: 5O3L), (c) Pick’s fold (PDB ID: 6GX5), and (d) identified binding modes for bTVBT4 in site A during MD simulation, with separation of down (D), up (U), left (L), and right (R) orientation of the ethyl group with respect to His362 (orange) and Ile360 (blue).

Separate unbiased MD simulations were run with 60 ligands inside and outside the cavity formed by the C-shaped protofilaments along the fibril axis. In Figure 3a, we present the combined ligand density for these simulations, where the density is averaged over time (200 ns) as well as summed along the fibril axis by overlaying all protofilaments on top of one another. From this density plot with maximum values color-coded in red, it is clear that the strongest ligand–protein interactions occur at two separate sites inside the cavity labeled A (higher density) and B (lower density).

In binding site A, bTVBT4 primarily interacts with residues Ile360, Thr361, and His362, and in site B, the ligand primarily interacts with Arg349, Val350, and Gln351; see Figure 3b. From the interaction energies presented in Table 2, it is clear that the ligand binding at site B is driven by Lennard-Jones interactions, whereas that at site A also has significant contributions from electrostatic interactions. In a recent experimental study, the tau binding sites for a ligand denoted APN-1607, were revealed with the cryo-EM technique.^34^ Common for the ligand structures of APN-1607 and bTVBT4 are the benzothiazole scaffold and vinylene moiety, and we note that the binding sites A and B found in our unbiased MD simulations are also identified in the cryo-EM maps but with reversed probabilities, *i.e.*, for APN-1607, site B is the major binding site, whereas site A is one out of several minor binding sites (see Figure 1e in ref (34)). This suggests that the bi-thiophene moiety steers the binding toward site A and exemplifies an exciting prospect of ligand binding control by chemical design. A theoretical docking study of several PET tau tracers belonging to different chemical families has been conducted,^35^ showing binding to site B but not site A and thus suggesting that bTVBT4 stands out in comparison to other ligands.

**Table 2:** Lennard-Jones (LJ) and Coulombic Interaction Energies (in kJ/mol) between bTVBT4 and Residues Ile360, Thr361, and His362 That Make up Binding Site A; and Arg349, Val350, and Gln351 That Make up Binding Site B^a^

site	type				total
A		Ile360	Thr361	His362	
	LJ	–50.5 ± 7	–4.8 ± 2	–36.7 ± 8	–91.9 ± 9
	Coulomb	4.8 ± 1	–1.8 ± 2	–24.9 ± 14	–21.9 ± 14
B		Arg349	Val350	Gln351	
	LJ	–73.2 ± 13	–13.0 ± 4.0	–32.0 ± 11	–118.2 ± 14
	Coulomb	5.6 ± 12	–7.8 ± 4	1.8 ± 9	–0.5 ±16

a Residue Arg349 has a charge of +*e*, whereas others are charge-neutral.

Focusing on the strongest binding site A, there are four major binding modes depending on the up/down (U/D) and left/right (L/R) orientation of the ethyl group with respect to the reference frame defined in Figure 3d. In addition, for each major mode, there are four minor modes associated with the dihedral rotations around the thiazole–ethyl bond and the bi-thiophene inter-ring bond, but the barriers in between these conformations are low, and interconversions occur frequently during the course of the dynamics; see Figures S6 and S12 in the SI.

**Figure 4:**
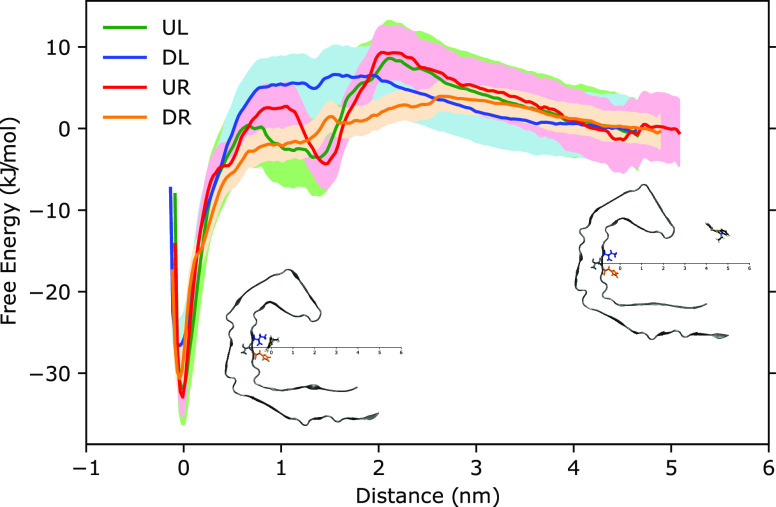
Free energy profiles for bTVBT4 in the UL (green), DL (blue), UR (red), and DR (orange) modes, obtained by the potential of mean force approach by pulling the ligand from binding site A to become free in solution.

To obtain the free energy profiles for the bTVBT4 binding in the four major modes of site A, we adopted the PMF approach in conjunction with umbrella sampling.^36^ The ligand was pulled from each of the major modes some 5 nm in the direction of the entrance of the cavity, as depicted in the two insets of Figure 4. At a pulling distance of about 1 nm, some of the trajectories resulted in interactions with site B, which is seen as shallow local minima on the potential. As exemplified by the PMF for mode DR, barrierless access to site A inside the cavity is available for bTVBT4. Further, we note that the binding energies are about 26–33 kJ/mol for the different modes with a mode ranking in terms of binding strength according to UR > UL > DR > DL, *i.e.*, up is favored over down and right is favored over left. Based on the binding energies calculated from this PMF study, the Boltzmann distribution of the major modes at site A are estimated to be 47, 30, 19, and 4%, respectively.

### Absorption Spectra of bTVBT4 in the Tau Binding Site

A correct spectrum averaging also requires the minor mode populations, and we obtained these by performing separate MD simulation runs for each of the four major modes (500 ns in each mode) and determining the times spent in the minor modes—a smaller model system based on 10 protofilament layers was used in these time-extended simulations. In the end, 300 uncorrelated snapshots with at least 20 ps separations were extracted from the simulations in accordance with the major and minor mode population statistics.

We determined the PE parameters needed for the spectrum calculations by means of the procedure of molecular fractionation with conjugate caps.^37^ For the Aβ(1–42) fibril periodic model system, it has been shown that the PE parameters determined in this manner can be considered to be time-independent, *i.e.*, not changing over the course of the dynamics as the fibril is in fact quite rigid.^27^ But, the ligand p-FTAA exhibits a strong Coulombic-driven binding to Aβ at fixed loci, while the bTVBT4 ligand shows movements along the fibril axis while remaining in binding site A (see Figure S13). We therefore approximated the PE parameters for tau not only to be time-independent but also unresponsive to the ligand interaction, *i.e.*, we determined a single set of protofilament parameters by averaging over 10 layers in the absence of a ligand and then adopted this set to all protofilaments and all snapshots—the alternative to derive snapshot-specific PE parameters was not pursued due to reasons of computational cost.

**Figure 5:**
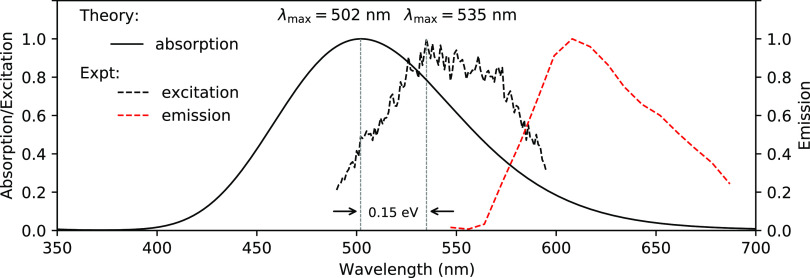
Theoretical absorption and experimental excitation and emission spectra for bTVBT4 bound to the tau protein in the Alzheimer fold. The 10 lowest states are included in the calculations performed at the level of TDDFT/CAM-B3LYP(100%). The experimental excitation and emission spectra were recorded at room temperature from tau deposits in a bTVBT4-stained AD brain tissue section washed with PBS (see SI section 2.2 for further details).

With a polarizable embedding of 20 Å and based on 300 snapshots to reach model convergence, the absorption spectrum of bTVBT4 in binding site A was obtained and is presented in Figure 5. The theoretical absorption spectrum has a λ_max_ of 502 nm, which represents a blue shift of 33 nm compared to the corresponding experimental excitation spectrum. Expressed in terms of energy, the discrepancy between theory and experiment amounts to 0.15 eV, and we deem this to be reasonable, given the nonspecific and fully classical description that had to be adopted for the protein environment.

The planarity parameter defined in ref (29) was shown to correlate well to the transition energy for anionic LCOs and be the key descriptor for the changes of optical responses of p-FTAA upon binding to Aβ.^27^ For bTVBT4, this parameter changes insignificantly from 3.43 ± 0.20 in solution to 3.49 ± 0.18 in site A of tau, suggesting that the associated red shift in the absorption spectrum is primarily due to changes in the electronic structure and not molecular structure. When comparing experimental decay times of bTVBT4 in various solutions and when bound to tau aggregates in AD, it is noted that it displays strikingly longer decay times in the latter case, ranging from 1.7 to 2.4 ns; see Figure S19 in the SI. Such increases in LCO decay times and also fluorescence intensities are believed to be associated with constrained vibrational motions,^27^ so although the difference in the theoretical planarity parameter is small for bTVBT4 in between solution and protein, there is experimental evidence that bTVBT4 adopts a more distinct conformation when bound to the aggregates.

**Figure 6:**
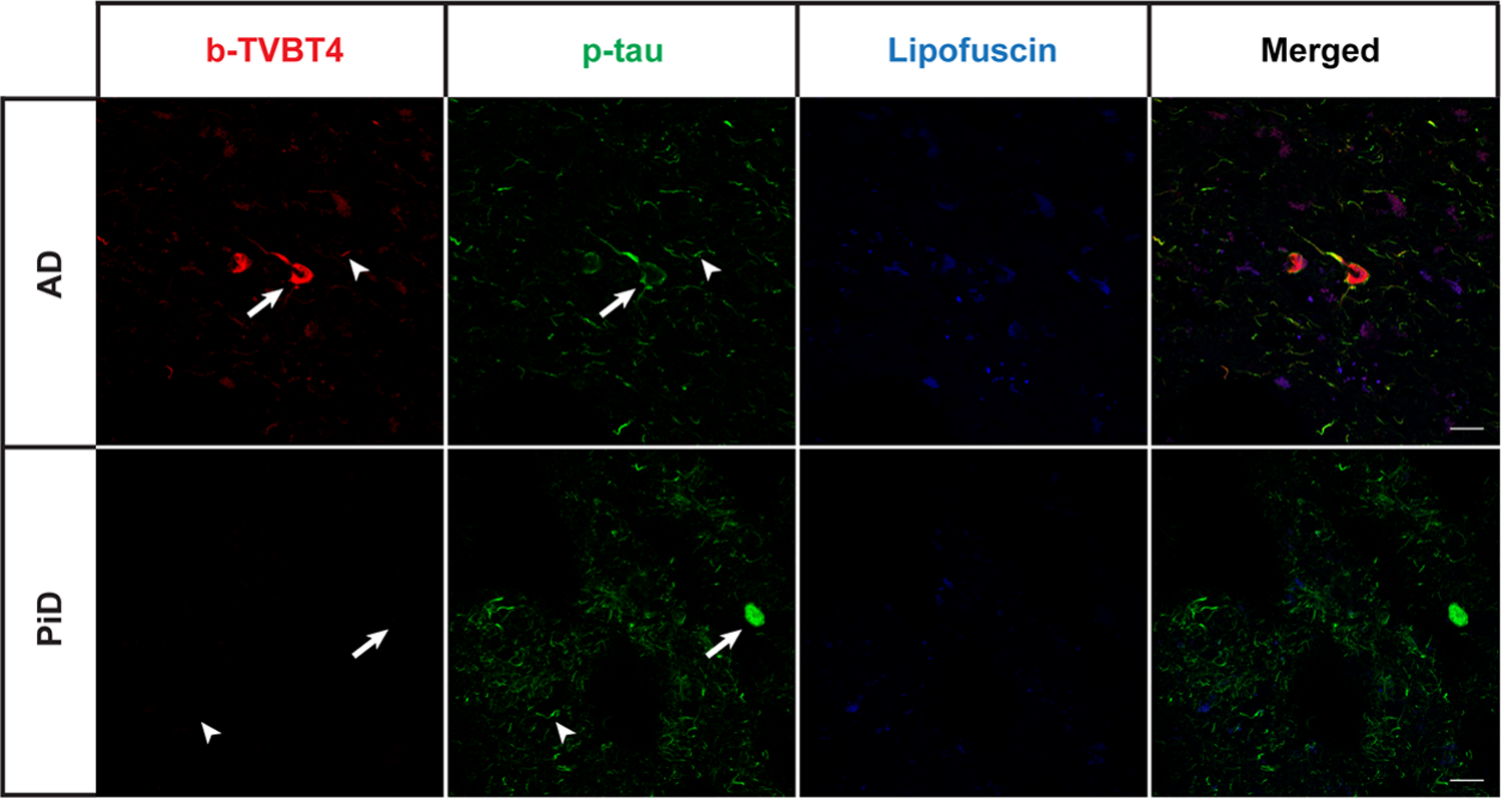
Fluorescence images of the brain section from a patient diagnosed with Alzheimer’s disease (AD, top panel) or Pick’s disease (PiD, bottom panel) stained with 100 nM bTVBT4 (red) and phospho-tau antibody AT8 (p-tau, green). Arrow: neurofibrillary tangle (AD), Pick body (PiD). Arrowhead: neuropil thread. As the autofluorescence from lipofuscin granules can overlap with bTVBT4 emission, an additional channel in which the settings only allowed excitation of lipofuscin (blue) is also shown. Scale bars represent 20 μm.

When comparing the cryo-EM structure for the tau fold in AD^2^ to that in Pick’s disease (PiD) fold,^38^ see Figure 3c, it is seen that the binding site A in tau is ligand-accessible in AD but not in PiD. Therefore, we next stained brain tissue samples from patients diagnosed with AD or PiD with bTVBT4 as well as an antibody reference marker AT8 that detects abnormally phosphorylated tau present in both AD and PiD;^39^ see column two in Figure 6. When stained with 100 nM bTVBT4, the AT8-immunopositive aggregates in AD, such as neurofibrillary tangles, neuropil threads, and dystrophic neurites, become visible through bTVBT4 fluorescence, whereas the AT8-immunopositive aggregates in PiD, such as Pick bodies, do not; see column 1 in Figure 6. The staining of tau deposits in PiD was also absent when using 10 times higher (1 μM) ligand concentration (data not shown). Hence, these tissue-staining experiments support our proposal of site A as the main tau binding site for the cationic bTVBT4 ligand because the disease-specificity of the ligand is thereby explained by the differences in protofilament folds in AD and PiD.

## Summary and Conclusions

A comprehensive theoretical–experimental study of the cationic luminescent ligand bTVBT4 has been carried out in the context of tau protein detection in the brain section from patients diagnosed with Alzheimer’s disease. The theoretical work includes (i) the development of a tau fibril model system based on the published cryo-EM structure,^2^ (ii) the methodological advancement for simulations of inhomogeneous vibronic absorption spectra of ligands in solution by a combination of an anharmonic vibrational theory and snapshot averaging, (iii) the identification of tau binding sites for bTVBT4 by means of unbiased atomistic molecular dynamics simulations, and (iv) the characterization of the strongest binding site by means of potentials of mean force and absorption spectra. The experimental work included (i) the determination of solvatochromic shifts in absorption and emission spectra, (ii) the determination of the excitation and emission spectra of bTVBT4 bound to tau, and (iii) the presentation of fluorescence images of the brain section from patients diagnosed with Alzheimer’s and Pick’s diseases as to address the specificity in the bTVBT4 binding. All things considered, there is strong evidence for the strongest interactions between bTVBT4 and tau to occur at the site involving residues Ile360, Thr361, and His362, and for the binding at this site to be predominantly driven by Lennard-Jones interactions.

## Supporting Information Available

Vibronic UV/vis spectra; details on the force field parametrization as well as MD, PMF, and spectrum simulations; ligand planarity analysis; and experimental details (PDF)
