# Supplementary material for: Tau Protein Binding Modes in Alzheimer’s Disease for Cationic Luminescent Ligands
Source: J Phys Chem B. 2021 Oct 13;125(42):11628–36. doi: 10.1021/acs.jpcb.1c06019 (PMC8558859; doi:10.1021/acs.jpcb.1c06019)
Supplement: Supplementary file 1 — jp1c06019_si_001.pdf [file jp1c06019_si_001.pdf]

Tau Protein Binding Modes in Alzheimer's Disease for Cationic Luminescent Ligands

Yogesh Todarwal,[†] Camilla Gustafsson,[†] Nghia Nguyen Thi Minh,[‡] Ingrid Ertzgaard,[¶] Therése Klingstedt,[§] Bernardino Ghetti,[‖] Ruben Vidal,[‖] Carolin König,[‡] Mikael Lindgren,[¶] K. Peter R. Nilsson,[⊥] Mathieu Linares,[†,#,@] and Patrick Norman[*,†]

†*Department of Theoretical Chemistry and Biology, School of Engineering Sciences in Chemistry, Biotechnology and Health, KTH Royal Institute of Technology, SE-106 91 Stockholm, Sweden*

‡*Leibniz University Hannover, Institute of Physical Chemistry and Electrochemistry, Callinstr. 3A, 30167 Hannover, Germany*

¶*Department of Physics, Norwegian University of Science and Technology, 7491 Trondheim, Norway*

§*Department of Physics, Chemistry and Biology, Linköping University, SE 581 83 Linköping, Sweden*

‖*Department of Pathology and Laboratory Medicine, Indiana University School of Medicine, IN 46202, USA*

⊥*Department of Physics, Chemistry and Biology, Linköping University, SE-581 83 Linköping, Sweden*

#*Laboratory of Organic Electronics, ITN, Linköping University, SE-581 83 Linköping, Sweden*

@*Scientific Visualization Group, ITN, Linköping University, SE-581 83, Linköping, Sweden*

E-mail: panor@kth.se

Supplementary Information

1 Theoretical methodology, computational details, and supplementary results

1.1 Modeling bTVBT4 biomarker properties in vacuum and solution

All geometry optimization calculations were performed in the Gaussian (version 16.B.01) program,[1] and all spectra calculations were done in the Dalton (version 2018.alpha) program.[2] The ground state geometry optimization of all the conformers (Fig. S1b) was carried out using B3LYP[3–5] density functional theory in conjunction with the 6-31+G(d,p)[6,7] basis set. A single point absorption spectra calculation was performed on each optimized geometry of the conformer using TDDFT with CAM-B3LYP(100%) [CAM-B3LYP ($\alpha = 0.19, \beta = 0.81, \mu = 0.33$)][8] functional in combination with aug-cc-pVDZ[9] basis set. The choice of basis set 6-31+G(d,p) for the geometry optimization and aug-cc-pVDZ for single point absorption spectra calculation was made to reduce computational cost, and in addition, as shown in our benchmark calculations summarized in Table S3, both basis set yield very similar values for absorption maxima for the $S_1 \leftarrow S_0$ transition. For the most stable conformers *cis* and *trans* (conformer 1 and 2 in the Fig. S1b), the ground state geometries was re-optimized at the B3LYP/aug-cc-pVDZ and dipole moment were calculated at the CAM-B3LYP(100%)/aug-cc-pVDZ method, keeping gauge-origin at the center of nuclear charge. The Fig. S2 shows the dipole moment vectors of trans conformer in the ground state (μ_{S_0}), excited state (μ_{S_1}) and their difference (μ_{diff}). The magnitudes of μ_{S_0}, μ_{S_1}, and μ_{diff} are 5.54 D, 2.08 D, and 4.74 D, respectively. The dipole moment of the first excited state is significantly lower as compared to the ground state. This decrease in dipole moment can also be seen from the NPA charge analysis shown in Fig. 1.

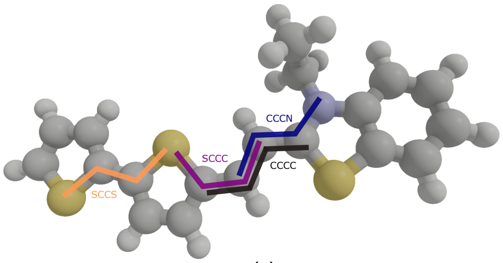

(a)

bTVBT4 geometry	SCCS	SCCC	CCCC	CCCN	ΔE (kJ/mol)	λ nm (eV)
1 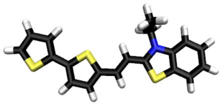	180	0	180	180	0.0	A: 472 (2.63)<br>B: 488 (2.54)
2 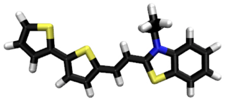	0	0	180	180	2.4	A: 472 (2.63)<br>B: 488 (2.54)
3 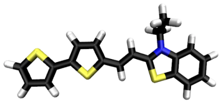	180	180	180	180	6.8	A: 479 (2.59)<br>B: 496 (2.50)
4 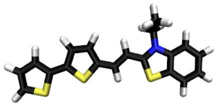	0	180	180	180	9.5	A: 479 (2.59)<br>B: 494 (2.51)
5 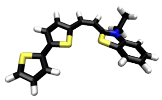	180	0	0	180	32.4	A: 477 (2.60)
6 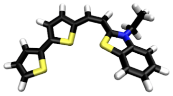	0	0	0	180	34.2	A: 473 (2.62)
7 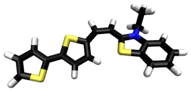	180	180	0	180	35.0	A: 490 (2.53)
8 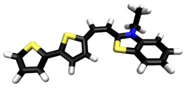	0	180	0	180	37.8	A: 488 (2.54)
9 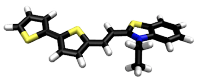	180	0	180	0	16.1	A: 468 (2.65)
10 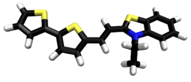	0	0	180	0	18.8	A: 468 (2.65)
11 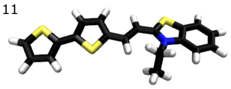	180	180	180	0	22.4	A: 473 (2.61)
12 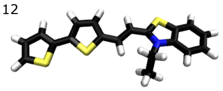	0	180	180	0	24.8	A: 471 (2.63)
13 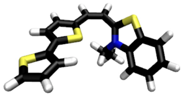	180	0	0	0	41.3	A: 440 (2.82)
14 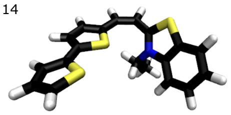	0	0	0	0	43.0	A: 436 (2.84)
15 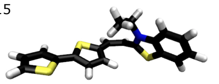	180	180	0	0	55.2	A: 421 (2.95)
16 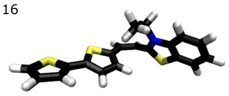	0	180	0	0	58.4	A: 418 (2.96)

(b)

Figure S1: (a) Naming convention for dihedral angles. (b) Possible conformations of bTVBT4. For simplicity in identifying conformer, dihedral angles between 0–90 degrees are denoted as 0, and angles between 90–180 degrees are denoted as 180. Relative energies are calculated with respect to most stable conformer. All structures were optimized at the B3LYP/6-31+G(d,p), followed by frequency calculation at the same level. All absorption spectra calculations were performed at two different levels: [A] CAM-B3LYP(100%)/aug-cc-pVDZ and [B] CAM-B3LYP/aug-cc-pVDZ , adopting the B3LYP-optimized geometries.

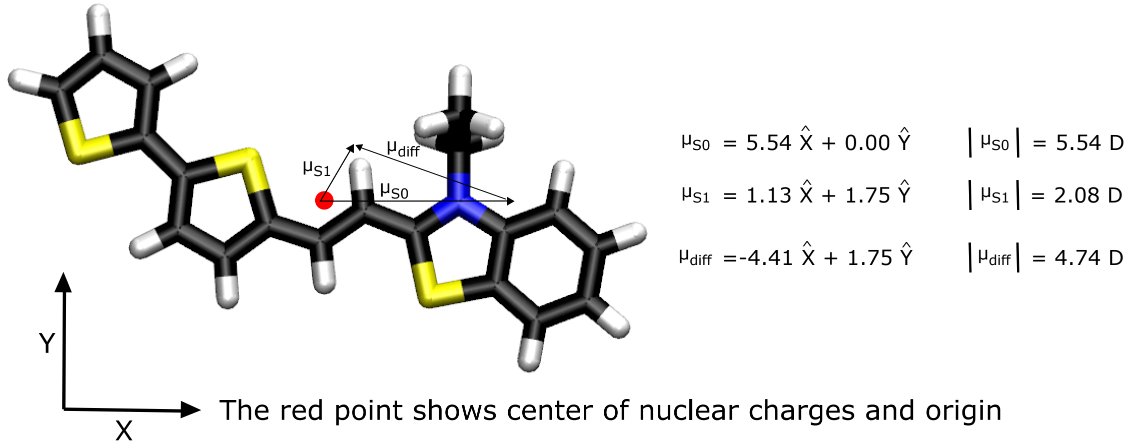

Figure S2: Calculated dipole moments of ground and excited state for *trans* conformer. Method: CAM-B3LYP(100%)/aug-cc-pVDZ (quadratic response theory, double residue) on ground state geometry optimized at B3LYP/aug-cc-pVDZ. Calculations are performed using Dalton. Symbols μ_{S_0}, μ_{S_1}, and μ_{diff} represent dipole moment of ground state, first excited state and difference between this two, respectively.

1.1.1 Vibrational structure and vibronic spectra

Several vibrational frequency calculations have been performed in order to aid in structural assignments at the harmonic level. The ground and excited state equilibrium structures for the *cis* and *trans* conformers were generated by TDDFT structure optimization in the first excited state using the long-range corrected and range-separated hybrid functional (CAM-B3LYP) and aug-cc-pVDZ basis set[10] in Gaussian package.[1]

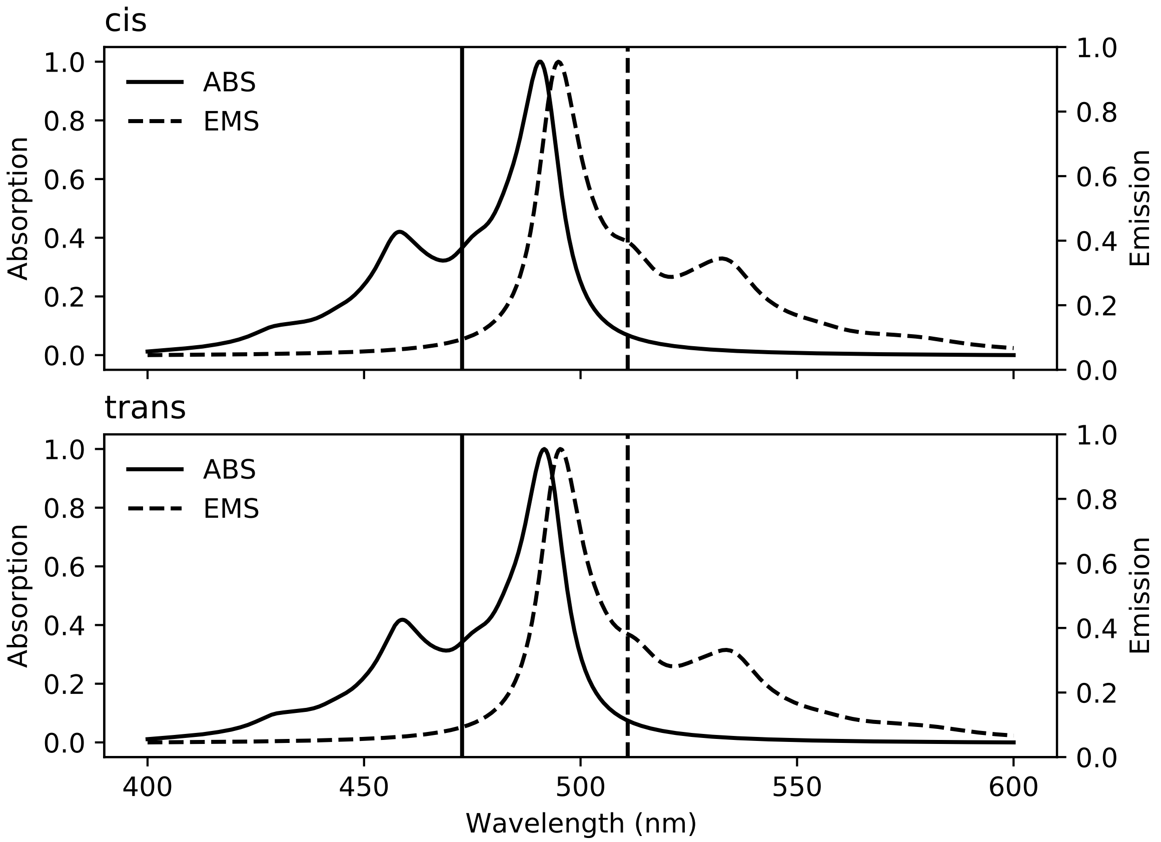

Figure S3: Computational vibrationally resolved absorption and emission spectra for *cis* and *trans* bTVBT4 in vacuum (CAM-B3LYP/aug-cc-pVDZ). For the *cis* reference structure, we included three modes (with the frequencies 1317 cm^{-1}, 1469 cm^{-1}, and 1600 cm^{-1}) anharmonically in the absorption calculation and six modes (646 cm^{-1}, 1246 cm^{-1}, 1428 cm^{-1}, 1458 cm^{-1}, 1466 cm^{-1}, and 1571 cm^{-1}) for the emission. For the *trans* reference structure, we accordingly treated three modes (i.e. 1323 cm^{-1}, 1468 cm^{-1}, and 1608 cm^{-1}) anharmonically in the absorption and seven modes (i.e., 680 cm^{-1}, 1215 cm^{-1}, 1241 cm^{-1}, 1427 cm^{-1}, 1459 cm^{-1}, 1482 cm^{-1}, and 1577 cm^{-1}) in the emission spectra calculation. All remaining vibrational modes were treated by the IMDHO model.

For generating the vibronic emission spectra, a hybrid scheme between the independent-mode displaced harmonic oscillator (IMDHO) model[11,12] and anharmonic treatment via vibrational configuration interaction (VCI) was employed. For the IMDHO we applied an in-house Python code and with half-widths at half-maximum (HWHM) of 200 cm^{-1}. For the anharmonic Franck–Condon factor calculations, we employed MidasCpp.[13] For the calculation of Franck–Condon factors in MidasCpp, see also ref. 14. In the construction of the potential energy surfaces (PESs), we made use of a multi-state[15] adaptive approach for grid point selection.[16] For expansion of the PES, we applied polynomials up to 8th order. No mode–mode couplings have been accounted for in the PES. The vibrational self consistent field (VSCF) calculations underlying the VCI calculations were performed using a b-spline basis.[17] 30 of the VSCF one-mode functions were considered in the following VCI calculations, which accounted for configurations with a maximum of three simulaneously excited modes (VCI[3]).

1.1.2 Molecular mechanics force field parameters of bTVBT4

Force field (FF) parametrization was carried out in two parts. In the first part, initial parameters were generated from the General Amber Force Field (GAFF).[18,19] In the second part, equilibrium bond distances, angles, and important dihedral potential were corrected based on optimized ground state geometry and dihedral potential calculated from DFT method.

Generation of initial parameters

To start with, rudimentary FF parameters of bTVBT4 were obtained from GAFF, following the steps outlined in an Amber tutorial,[20] summarized below.

- Step 1: Geometry Optimization

  The initial geometry of bTVBT4 molecule is optimized in the Gaussian program by employing the B3LYP functional and 6-31+G(d,p) Pople's basis set.

- Step 2: Restrained Electrostatic Potential (RESP)[21] charge calculation and defining

atom types

In Amber FFs, all residues (building blocks) on which macromolecules are built are restricted to integer charges. Having integer charges on building blocks help in transferring electrostatic parameters to macromolecules. RESP charges assure that rotationally degenerate atoms, such as hydrogens of methyl and methylene have equivalent charges. Determining the RESP charges and atom type involves; A) Calculating the electrostatic potential (ESP) at HF/6-31G* on an already optimized geometry in gas phase. B) Fitting charges at each nucleus by minimizing the sum squared error between calculated ESP from charges and ESP obtain from step 2A. This is achieved with the help of Antechamber (part of Amber tools).[18,19]

- Step 3: Parameter check and building a parameter library

  Once the atom types are defined, a parameter library for bTVBT4 was generated using LEaP (part of Amber tools).

- Step 4: Format conversion

  The topology obtained from the above procedure is in Amber format, and since we will be using Gromacs[22–25] for molecular dyanmics (MD), the topology is converted from Amber to Gromacs format using the ACPYPE[26] Python module.

Correcting bond distances, bond angles and dihedral angles

During step 3 described above, the parmchk program (part of Amber tools) has created suggestions for some dihedrals which was not found in the GAFF database. We are interested in determining the dynamics of bTVBT4 in different environments and calculating an absorption spectrum on frames sampled from the dynamic trajectory. Hence, it is important to accurately describe bond distances, bond angles, and dihedral angles so that absorption spectra calculated on DFT and Molecular Mechanics (MM) optimized geometry give same result. In order to obtain more suitable GAFF parameters and validate the FF, the following steps were taken:

- Step 1: Dihedral scanning

  A relaxed dihedral scan for each of the dihedral angles defined in Fig. S1(a) was performed using B3LYP/6-31+G(d,p) as well as Molecular Mechanics (MM) with the GAFF. Comparing the two (DFT and MM) potential energy curves in Fig. S4 (illustrating the SCCS dihedral angle), we see that the initial topology that was generated is not an adequate description of SCCS potential. Due to the artificially high barrier represented in GAFF, an interconversion between the *cis* and *trans* conformation of bTVBT4 gets hindered during the MD simulation. Similarly, it is important to represent other important dihedral angles SCCC, NCCC, and CCCC accurately, in order to sample conformation at the 300 K.

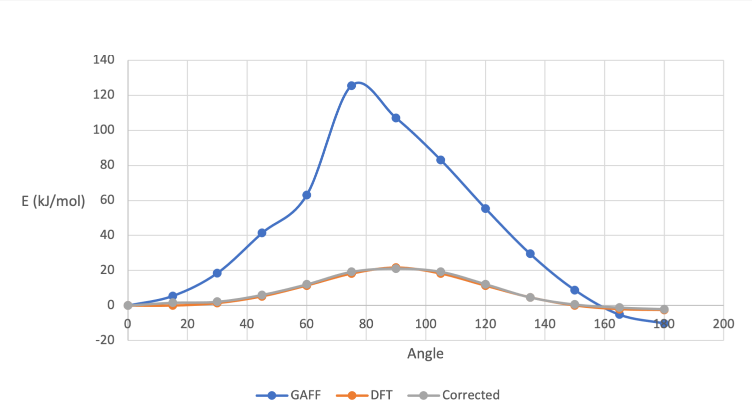

Figure S4: Dihedral potential of SCCS (marked in Fig. S1(a) of bTVBT4) from GAFF (blue), DFT (orange), and corrected FF (grey).

- Step 2: Modifying equilibrium bond distance and bond angle.

  Bond distances and bond angles are crucial for absorption spectra, so all equilibrium bond distances and bond angles from GAFF were replaced with values obtained from DFT optimized geometries. All remaining parameters were kept the same as obtained from GAFF.

- Step 3: Excluding the energy contribution from dihedral of interest

  For the required dihedral, first, a contribution of torsion energy is excluded from total

MM energy by setting appropriate torsion parameters to zero in a FF file. Using this modified topology, MM based relaxed dihedral scan (called MM_zero) is obtained. Next, this MM_zero energy profile is subtracted from DFT energy profile to get a missing energy contribution which will be added to torsion parameters of dihedral of interest. For the SCCS dihedral, the difference between DFT and MM_zero curve is shown in Fig. S5 with red colour. Next task is to fit this curve with the Ryckaert-Bellemans function Eq. (1)).

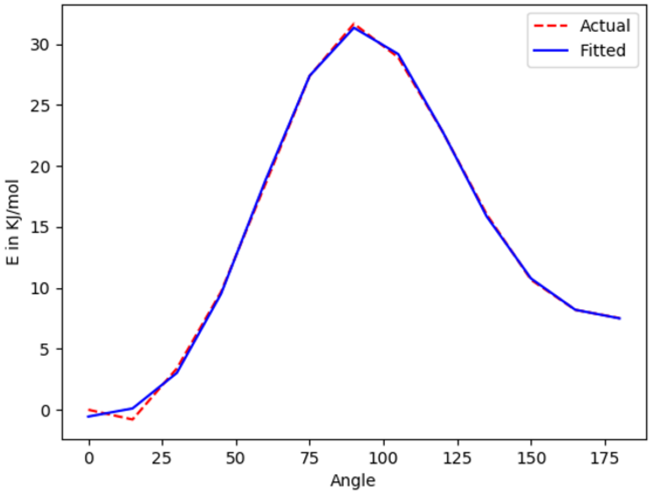

Figure S5: Curves shows the difference between potentials for DFT and MM_zero (where all parameters that define SCCS are substituted to zero).

- Step 4: Fitting to DFT potential

  The curve obtained by taking the difference between DFT and MM_zero potential is fitted with Ryckaert-Bellemans function (Eq. (1)). This function has previously been used to fit dihedrals involved in bi-thiophene based ligands.[27] Curve fitting was done using the Scipy[28] Python module. The fitted curve is shown in blue in Fig. S5.

$$V(\phi) = \sum_{n=0}^{5} C_n \cos^n(\phi - 180^\circ) \tag{1}$$

- Step 5: Adding corrected parameters and scanning the dihedral angle

  Add corrected parameters for dihedral which was fitted and a relaxed dihedral scan

was performed using this modified topology. The result obtained in this step for the SCCS dihedral is shown in Fig. S4 (grey curve). The same procedure was employed for correcting all dihedrals (SCCC, NCCC, and CCCC, illustrated in Fig. S1a) iteratively.

Note: So far in the process of fitting dihedral potential, except for the step 2 (correcting the bonds and angles based on DFT optimized geometry) we followed the procedure which has been previously described in Ref. 29.

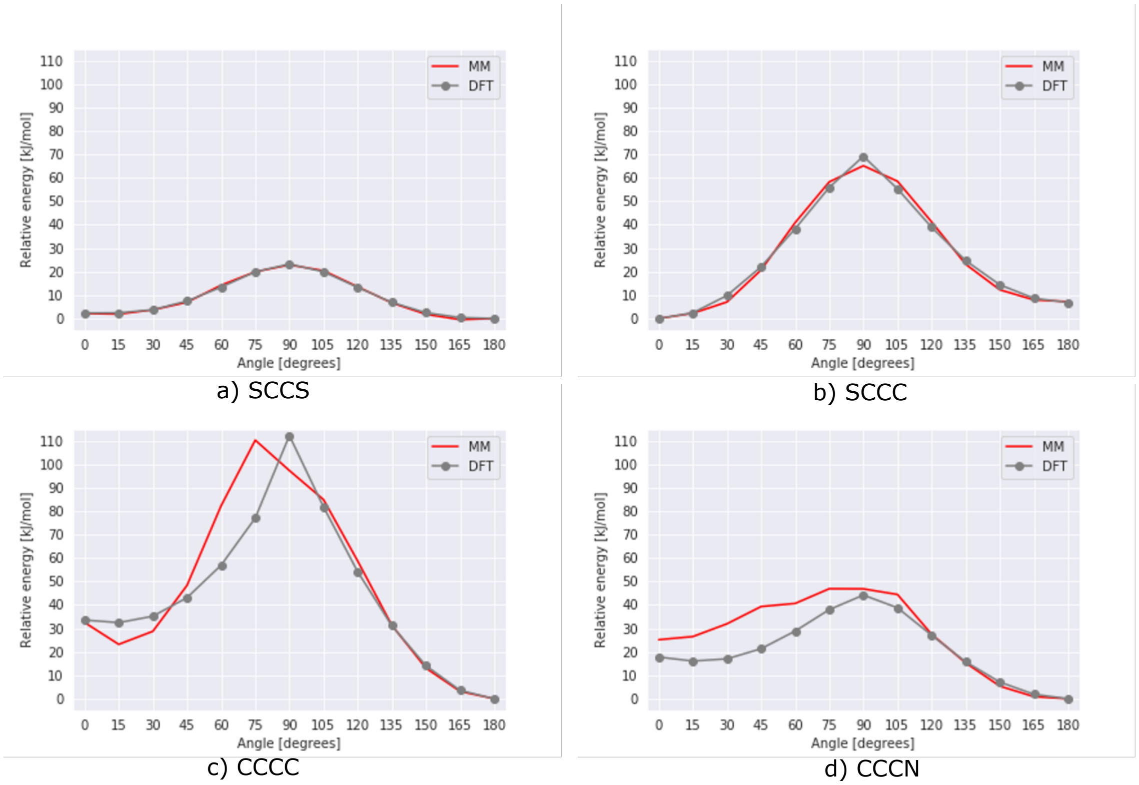

a) SCCS b) SCCC c) CCCC d) CCCN

Figure S6: Fitting of important dihedrals (marked in Fig. S1(a) of bTVBT4 to the DFT potential. The dihedral scans are performed at B3LYP/6-31+G(d,p).

- Step 6: Validation

  After correcting all important dihedrals, the final dihedral scan is shown in Fig. S6. In all the cases, the dihedral potential curve is in good agreement with DFT. For the energy barrier above 40 kJ/mol, the dihedral potential of the CCCN dihedral deviate from DFT potential, however this will not be the problem in current study as simulations are performed at room temperature (300 K). Next, if we compare the

relative energies of *cis* and *trans* obtained using different methods (DFT and the re-parametrized FF), it is in good agreement (Table S1). This can also be seen from Boltzmann distribution shown in Table S2.

Table S1: Comparison of QM vs. MM ground state energy difference for the most stable (*cis*/*trans*) bTVBT4 conformers.

Method	$\Delta E_{cis-trans}$ kJ/mol (kcal/mol)
QM (B3LYP/6-31+G(d,p))	2.47 (0.59)
QM (B3LYP/aug-cc-pVDZ)	1.97 (0.47)
MM (Modified GAFF)	2.22 (0.53).

Table S2: Comparison of QM vs. MM Boltzmann distribution for ground-state conformations.

Method	$\Delta E_{cis-trans}$ kcal/mol	P_{cis}	P_{trans}
QM (B3LYP/6-31+G(d,p))	0.59	0.27	0.73
MM (Modified GAFF)	0.53	0.29	0.71

- Step 7: Absorption spectrum test

  Absorption spectra calculated on *cis*/*trans* geometries optimized using the re-parametrized FF are presented in Table S3, along with transition wavelengths computed at different levels of DFT. The transition wavelengths calculated using MM optimized geometries were in very good agreement with DFT values.

Table S3: Comparison of QM vs MM ground state absorption spectra of bTVBT4 conformations in vacuum, calculated at TDDFT/CAM-B3LYP(100%)/aug-cc-pVDZ using Dalton program.

Geom. opt. method	*cis* nm (eV)	*trans* nm (eV)
QM B3LYP/6-31+G(d,p)	471.7 (2.628)	472.3 (2.625)
QM B3LYP/aug-cc-pVDZ	473.4 (2.619)	472.9 (2.622)
MM (Modified GAFF)	471.3 (2.631)	472.4 (2.625)

1.1.3 Functional benchmark for absorption spectra

Generally, changing the DFT functional induce a global shift in transition energies and does not influence the absorption spectrum profile. In order to have computational transition en-

ergies close to experimental values, a choice of functional was based on benchmark calculation shown in Table S4.

Table S4: Functional benchmark for absorption spectra, calculated on B3LYP/6-31+G(d,p) optimized geometry of *trans* conformer.

Functional	*trans* nm (eV)
CAM-B3LYP(100%)/aug-cc-pVDZ	472 (2.625)
CAM-B3LYP/aug-cc-pVDZ	490 (2.532)
B3LYP/aug-cc-pVDZ	518 (2.395).

1.1.4 Molecular dynamics simulation

All classical MD simulations were performed using GROMACS (version 19.3)[22–25] with the Amber ff14SB force field for the tau protein,[30] TIP3P for water solvents,[31] and re-parametrized GAFF for bTVBT4. In particular, all MD processes (energy minimization, equilibration, and production) were performed for different types of systems: bTVBT4 in a water solvent, periodic tau fibril in water solvent (with and without bTVBT4), bTVBT4 in small tau fibril model. The solvation box was neutralized with counter-ions (Cl^-) for each of these models and described with the Amber forcefield (ff14SB). The protonation state of all amino acid residues of the tau protein are represented at the physiological pH of 7.4.

When the simulation is performed in the NPT ensemble (equilibration step), the reference temperature of 300 K was kept constant using the velocity rescaling weak coupling scheme with a coupling constant of $\tau = 0.2$ ps. A Berendsen barostat with a coupling constant of 1 ps was employed to maintain the pressure at 1 atm. In the case of bTVBT4 in tau protein, Berendsen barostat was used under semi-isotropic conditions. Whereas for bTVBT4 in water, the isotropic condition was applied. Periodic boundary conditions were applied to all three directions of the simulated box. Electrostatic interactions were simulated with the Particle Mesh Ewald (PME)[32] approach using a long-range cutoff of 1.5 nm. The cutoff distance of Lennard-Jones (LJ) interactions was also equal to 1.5 nm. The MD simulation time step was 2 fs with a pair-list update period of 10 steps. All H-bond lengths were kept

constant using the LINCS routine.[33]

1.1.5 Molecular dynamics simulation of bTVTB4 in vacuum

The ligand bTVBT4 (with one Cl^- counter ion) was first simulated in vacuum, and *cis* and *trans* conformations were sampled during a 4 μs long MD simulation. Percentages of *cis* and *trans* conformations of bTVBT4 are presented in Table S5. Initially, the Cl^- ion was positioned at 24 nm from bTVBT4. It moves closer (< 4 Å) to bTVBT4 during the trajectory, and starts interacting with bTVBT4 from about 98 ns and stays close to the ligand during the remainder of the simulation. Due to this fact, the expected percentage of *cis* and *trans* conformations from Boltzmann distribution is not achieved.

Table S5: Percentage of *cis* and *trans* conformations from 4 μs long MD simulation of bTVBT4 and Cl^- ions in vacuum.

	cis	*trans*
Percentage from MD simulation	64	36
Percentage from Boltzmann distribution	71	29

1.1.6 Molecular dynamics simulation of bTVTB4 in water

One bTVBT4 molecule in *trans* conformer was solvated by a water solvent box of $5.2 \times 5.2 \times 5.2$ nm^3. One Cl^- ion was added to neutralize the system. Next, the equilibration step was performed with the first energy minimization of the whole system, followed by a short 100 ps NPT simulation to stabilize the pressure of the system. Subsequently, we propagated the system in an NVT ensemble for the production run with accumulated time of 4 μs from 4 independent simulations.

The converged dihedral distribution over snapshots extracted from 4 μs data is shown in Fig. S7. The time step between each saved snapshot was 10 ps. The calculated percentage of *cis* and *trans* conformers of bTVBT4 in water are 50.5 and 49.5, respectively. Although, *trans* conformation that is the most populated in vacuum, the *cis* conformer is estimated

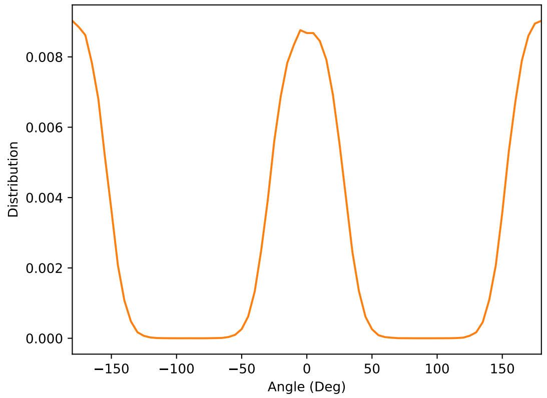

Figure S7: Dihedral SCCS (marked in Fig. S1(a) of bTVBT4) distribution in 4 μs long MD of bTVBT4 in water.

Table S6: Percentage of *cis* and *trans* conformations from a 4 μs MD simulation of bTVBT4 and Cl^- in water. LJ and Coulomb interaction energies of bTVBT4 with water are given in kJ/mol.

	cis	*trans*
Percentage	50.5	49.5
LJ	-135.9 ± 10	-136.3 ± 10
Coulomb	-68.3 ± 14	-62.1 ± 4

to be equally populated in water due to an increase in interaction energy with water. The decomposition of interaction energy shows that the LJ interaction energy is approximately equal for both the conformers, as presented in Table S6. However, the Coulomb interaction of *cis* conformer in water is more stable than *trans* conformer by 6.2 kJ/mol. This increase in Coulomb interaction energy is attributed to interaction between thiophene moiety of *cis* and water.

1.1.7 Polarizable embedding and spectra in solution

In order to include the effect of the water environment while calculating the spectra of bTVBT4, we employed the method of Polarizable Embedding (PE)[34,35] on the snapshots extracted from the MD simulations. PE is based on a hybrid quantum-classical computational approach in which the total system is split into three regions: core, polarizable, and

non-polarizable. The core region having only a bTVBT4 molecule is calculated in the quantum chemical description. The polarizable region is described with Ahlström charges and isotropic polarizabilities,[36] while the non-polarizable region is described with just TIP3P charges. The shell thickness of 15 Å polarizable and 5 Å non-polarizable regions are considered to be reasonable to account for the effect of water. The Fig. S8 shows the test calculation of transition wavelength as a function of shell thickness of polarizable (red), non-polarizable (blue), and combination of both (green).

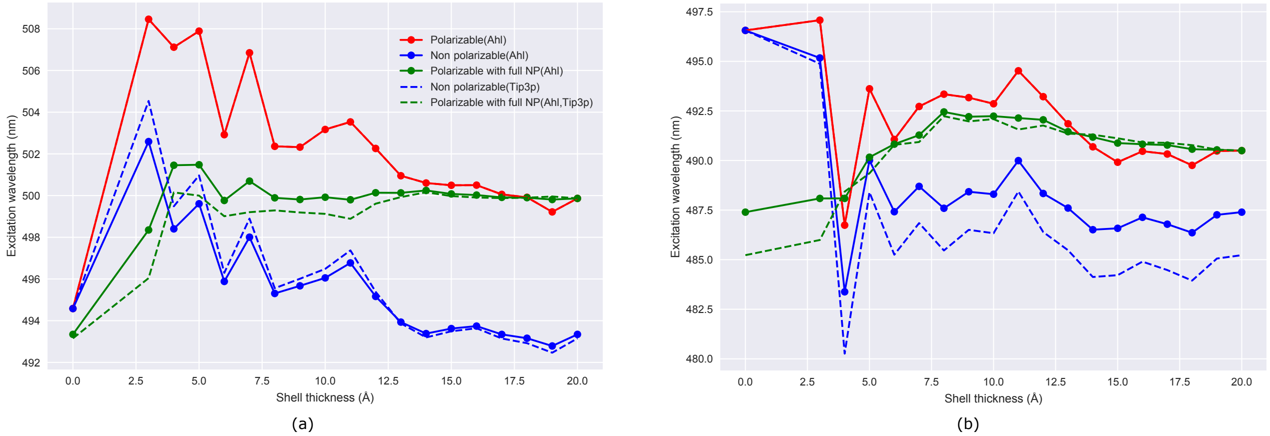

Figure S8: Transition wavelength of bTVBT4 as a function of shell thickness of polarizable (red), non-polarizable (blue), and polarizable with full NP (green) for (a) trans, and (b) cis snapshot extracted randomly from the simulation trajectory. The bold lines indicate when the non-polarizable region is represented by Ahlström charges and the dashed lines when the TIP3P charges are used. The absorption spectra calculation are performed in CAM-B3LYP/aug-cc-pVDZ.

From the MD simulation, a total of 200 number of snapshots were extracted of which in 100 frames bTVBT4 was in *cis* conformation and for another 100, in *trans* (respecting the *cis*:*trans* ratio found in the MD simulation, see Fig. S7), while ensuring converge spectral profile. The time step between two consecutive frames was at least 10 ps. For each snapshot, single point absorption spectra calculation was performed where the bTVBT4 was described in a QM core, all atoms within 15 Å of bTVBT4 with polarizable and following 5 Å shell in the non-polarizable region. The calculations were carried out for the ten lowest excited states at the CAM-B3LYP(100%) and aug-cc-pVDZ basis set using the Dalton program.

Subsequently, the calculated absorption spectrum on each snapshot was Gaussian convoluted with standard deviation of 0.15 eV and also with line broadening that was obtained from vibrationally-resolved electronic spectra of bTVBT4 in vacuum. The final spectral profile was obtained by taking an average over all the snapshots under consideration see in article Fig. 2(b). For the set of 100 *trans* conformer, average absorption maxima is at 480 nm (2.58 eV). Whereas for the set of 100 *cis* conformer, absorption maxima is at 485 nm (2.56 eV), see Table S7.

Table S7: Calculated transition wavelength averaged over 100 *cis*/*trans* snapshots for bTVBT4 in water.

	trans	*cis*	average
Transition wavelength nm(eV)	480 (2.58)	485 (2.56)	482.5 (2.57)

One snapshot from the set of 100 *trans* frames that had a peak at the average maxima at around 480 nm was selected for further studies on the absorption spectra. On this particular snapshot, the QM region was increased to also encompass 5 water molecules in addition to the bTVBT4 molecule. The effect of this was only a negligible shift in the absorption spectrum (Table S8). However, increasing QM region further to accommodate 14 water molecules, the absorption maxima shifted by 5 nm from 480 nm (average value) towards the experimental value (470 nm). Nevertheless, this improvement comes at a large computational cost and therefore, we decided against including water molecules in the QM region.

Table S8: Further improvement in absorption spectra of bTVBT4 in water, by increasing the QM region. One *trans* snapshot having absorption maxima at 480 nm was selected for test calculation

Method	Transition wavelength nm (eV)
QM(bTVBT4)	480 (2.58)
QM (bTVBT4) + PE	480 (2.58)
QM (bTVBT4 + 5 H2O) + PE	479 (2.59)
QM (bTVBT4 + 14 H2O) + PE	475 (2.61)
Experimental	470 (2.64)

1.2 Modeling bTVBT4 biomarker interactions with tau fibril

1.2.1 Tau fibril in cryo-EM structure

Formation of amyloid and tau protein fibrils are the main characteristics of Alzheimer's disease. Fitzpatrick *et al.*[37] deduced the cryo-EM structure of tau protein. It was found to exists in two ultrastructural polymorphs: paired helical filaments (PHFs) and straight filaments (SFs). They differ in an inter-protofilament arrangement. The core of the structure (protofilament) is made up of 73 amino acids. In the case of PHFs, the two protofilaments are bound by 2_1 screw symmetry to form a monomer. These monomers stack on each other to develop a long fibril structure. Consecutive monomers along the protofilament have an approximate twist of 1 degrees and distance 4.7 Å.

1.2.2 Creating a tau fibril models

From the PDB ID 5O3l, first, coordinates of one protofilament out of ten were extracted. Next, by taking the 2_1 screw symmetry of the first protofilament, another protofilament was generated to form a monomer. The consecutive monomers were obtained by rotating a previous monomer by 0.97 degrees and translated it by 5 Å. This operation was continued until the last monomer had a twist of 180 degrees with respect to the first monomer. It is important to ensure a perfect fit between the last monomer and the first monomer of an adjacent periodic image of the simulation box. In this way a long fibril was modeled, which approximately resemble the experimentally found tau fibril while decreasing the computational cost by half. Employing the same strategy, tau models having adjacent monomer twist of 0.8, 0.9, 1.0, ad 1.2 were also constructed to find a fibril model with least stress induce due to forced periodicity.

1.2.3 MD simulation of full tau fibril

For each of the tau model created, simulated annealing was performed to slowly warm the system under NPT conditions. The heating of system from 150 K to 300 K was achieved with 14 annealing points, at the temperature step size of 25 K.

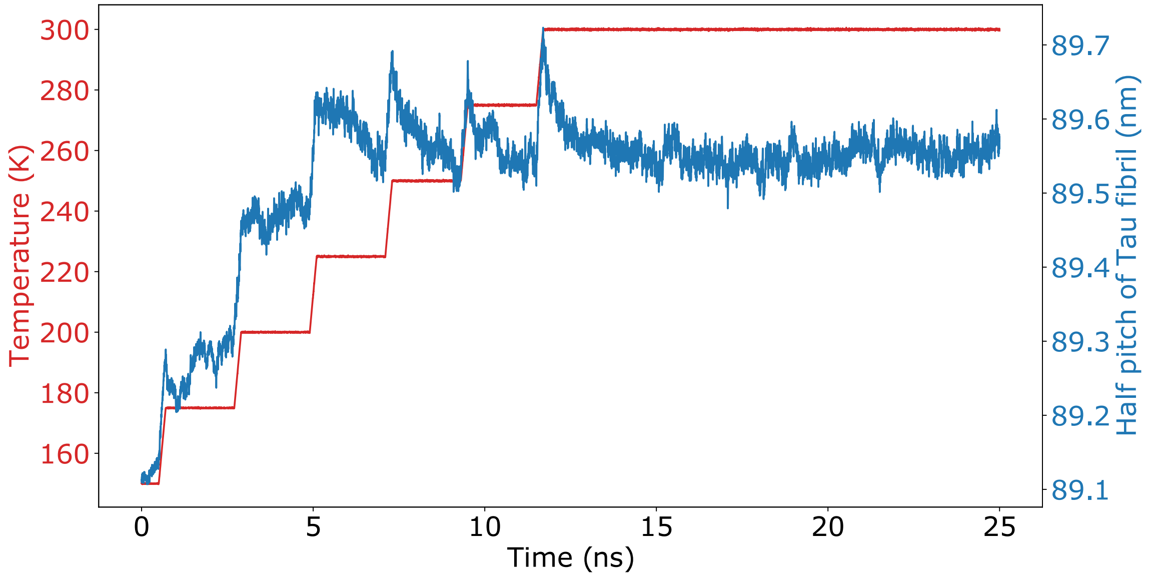

Figure S9: Length of half pitch tau fibril (blue) as a function of temperature (red) during 25 ns simulated annealing MD.

The temperature of the system was gradually increased every 200 ps, and at each step, it was simulated for 2 ns except for the last step of 300 K where it was simulated for 13 ns. It was observed that tau fibril generated with the adjacent monomer twist of 0.97 degrees had fewer kinks compared to the other tau models with different adjacent monomer twits (data not shown). The dynamics of the total fibril length along its axis is shown in the Fig. S9 for the tau model with monomer twist of 0.97 degrees. Simulation at the final step of 300 K shows that we have reached an equilibrium structure from 14 ns onward. In the equilibrium structure of the tau model, the average distance between two adjacent monomers was decreased from 5 Å to 4.8 Å.

1.2.4 Molecular dynamics of full periodic model of tau with 60 bTVBT4 molecules

Starting from the thermally stable structure of the tau fibril model, two kinds of system were built to increase the sampling in MD simulation. In the first system, the simulation box contained the tau fibril with 60 bTVBT4 molecules inside the tau cavity (Fig. S10a), and in the second system, 60 bTVBT4 outside the tau cavity (Fig. S10b). Each ligand was placed at random position and in random orientation.

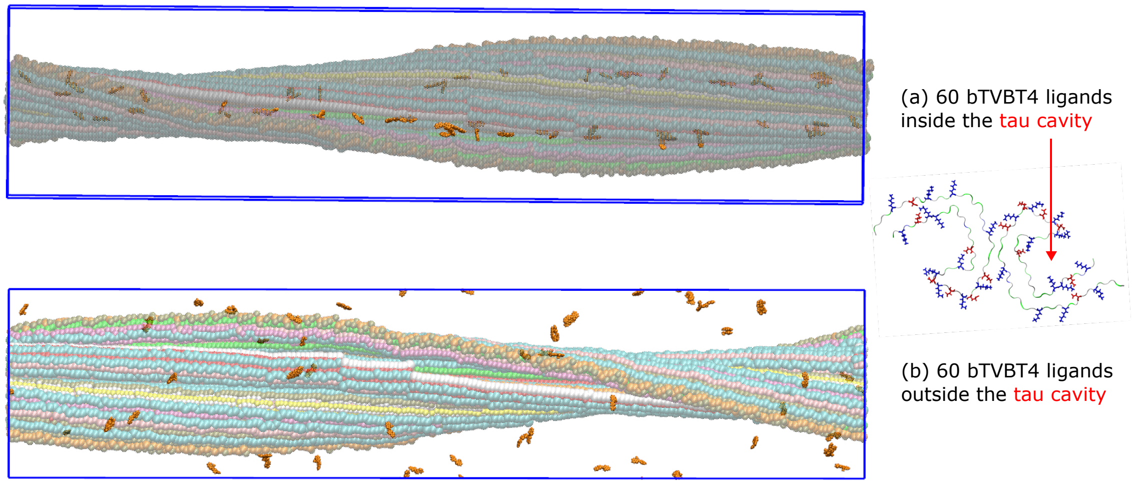

Figure S10: (a) 60 bTVBT4 ligands placed inside the cavity of tau fibril. (b) 60 bTVBT4 ligands placed outside the cavity of the tau fibril.

Initially, in the process of equilibration, both kinds of systems were energy minimized, followed by short 100 ps NVT simulation with position restrain on bTVBT4 and tau fibril to stabilize the temperature. Next 100 ps short NPT simulation to stabilize the pressure. Finally, the MD simulation production run was carried out in NVT ensemble, maintaining the temperature at 300 K. For the 60 bTVBT4 molecules inside tau cavity case, five independent simulations were simulated for 40 ns each, whereas for the 60 bTVBT4 molecules outside tau cavity case, four independent simulations were setup and simulated for approximately 35 ns each.

Binding mode code assignment for Site A :

Site A was distinguished as the strongest binding site at which bTVBT4 binds in various modes (see Fig. 3). These modes were characterized with respect to position of the ethyl

group, bi-thiophene group of bTVBT4 and amino acid residues Ile360, and His362 of the tau fibril that bTVBT4 interact with.

Modes are defined by four letters assigned based on the following criteria:

Fix the position of tau such that Ile360 is on top and His362 on the bottom side.

- Based on relative position of ethyl group with Ile360 and His362 assign "U" (ethyl pointing towards Ile360) or "D" (ethyl pointing towards His362)
- ethyl group is at left "L" or right "R" (keep position of Ile360 and His362 fixed as mention above)
- Bi-thiophene moiety in *cis* "C" or *trans* "T" conformation.
- CH_3 group on ethyl, inside "I" (pointing towards tau protein) or outside "O" (away from tau protein)

Considering all possible combination, in total there are 16 modes. The numeric and alphabetic codes for all 16 modes are defined in the Table S10. Frequency of interconversion between "I" , "O" and "C" , "T" is grater as compared to "U" , "D" and "L" , "R". Hence, when only the first two letters of alphabetic code are considered, we define it as major mode and when all four letters are considered we defined it as minor modes (see Fig. S11).

Table S9: Lennard-Jones (LJ) and Coulombic interaction energies (in kJ/mol) between bTVBT4 and residues Ile360, Thr361, and His632 that make up binding site A, and Arg349, Val350, and Gln351 that make up binding site B. Residue Arg349 has a charge of $+e$, whereas others are charge neutral.

Site	Type				Total
A		Ile360	Thr361	His362	
	LJ	−50.46±7	−4.78±2	−36.67±8	−91.9±9
	Coulomb	4.82±1	−1.76±2	−24.91±14	−21.85±14
B		Arg349	Val350	Gln351	
	LJ	−73.23±13	−13.01±4.0	−31.96±11	−118.21±14
	Coulomb	5.60±12	−7.84±4	1.79±9.	−0.45±16

Code	Minor mode	Major mode	Front view	Top view
12	ULTO	UL	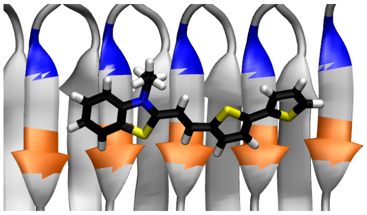	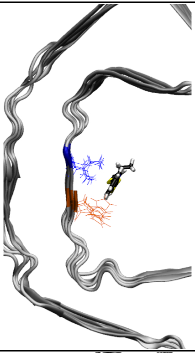
4	DLTO	DL	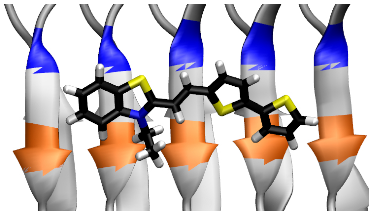	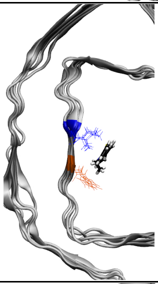
16	URTO	UR	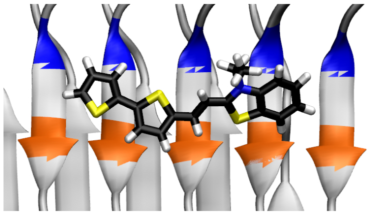	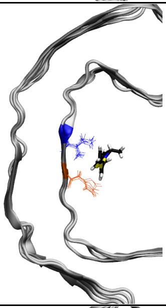
7	DRTI	DR	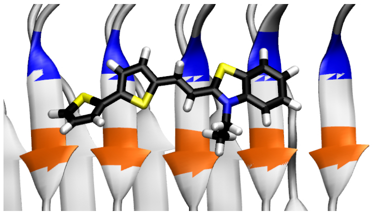	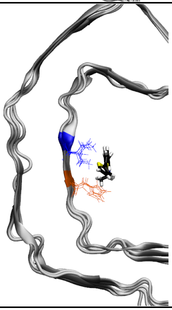

Figure S11: Examples of naming convention. The alphabetic code up/down (U/D) and left/right (L/R) are based on relative position of ethyl group with respect to residues Ile360 (marked in blue) and His362 (marked in orange). The *cis*/*trans* (C/T) is with respect to the dihedral between bi-thiophene moiety and inside/outside (I/O) based on the dihedral rotations around the thiazole–ethyl bond.

Average total interaction energy of bTVBT4 with site A:

From an MD simulation of the full tau model with 60 bTVBT4 molecules, two binding sites

were identified A (Ile360, Thr361, His362) and B (Arg349, Val350, Gln351) as seen in article Fig. 3. The LJ and Coulombic interaction energies of bTVBT4 in the binding site A and site B are shown in the Table S9. These energies were calculated only for those bTVBT4, which bound to the site (A or B) continuously for 20 ns and had LJ < -70 kJ/mol. The result shows that the ligand binding to site A is driven by Coulombic interaction while to site B by LJ interaction.

Table S10: Average of interaction energy (LJ + Coulomb) and number of bTVBT4 molecules at each mode. Energies are given in kJ/mol.

code	Mode	Number of molecules	Average energy	Standard deviation
1	DLCI	8	-160.5	19
2	DLCO	7	-157.3	17
3	DLTI	8	-157.7	18
4	DLTO	16	-151.5	17
5	DRCI	14	-160.5	21
6	DRCO	9	-156.0	19
7	DRTI	23	-154.4	18
8	DRTO	12	-150.4	17
9	ULCI	6	-147.5	18
10	ULCO	5	-151.3	16
11	ULTI	9	-144.8	15
12	ULTO	10	-150.8	20
13	URCI	2	-142.9	17
14	URCO	3	-153.0	16
15	URTI	10	-156.4	18
16	URTO	19	-147.0	17

The majority of the bTVBT4 population is found at site A at which it binds in 16 different minor modes (or 4 major modes) as defined in Table S10. The average total interaction energy (LJ+Coulomb) between bTVBT4 molecules and tau fibril calculated for each of the minor modes from the five independent MD simulations are shown in Table S10. Often interconversion from one minor mode to another is observed in the binding pocket during the trajectory, mainly between minor modes of type $XXTX \leftrightarrow XXCX$ and $XXXI \leftrightarrow XXXO$, where X represent any other letter codes for minor modes as defined in Table S10. The Fig. S12 shows all the interconversion between the minor modes. The average of the

total interaction energy over all minor modes is calculated to be -153 ± 19 kJ/mol. The bTVBT4 interaction with the site A residues account for 75% of the average total interaction energy when it interacts with the whole tau, as shown in Table S11.

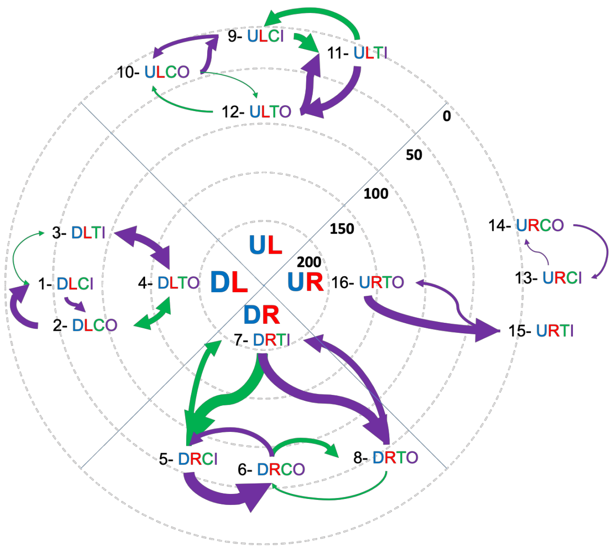

Figure S12: Exchange between minor modes at site A in the system of 60 bTVBT4 in the cavity of full tau model. Radius represent the time spent (ns) in each mode (accumulated time for all molecules in the mode), the thickness indicates the number of exchanges (largest 14).

Table S11: Comparison of bTVBT4 average total interaction energy (in kJ/mol) with protein and site A residues.

	with site A residues	with protein
bTVBT4 interaction	-114 ± 17	-153 ± 19

1.2.5 Smaller binding site model

The smaller binding site models were generated from the system of 60 bTVBT4 molecules in the cavity of the tau fibril by cropping out the four major binding modes. Each smaller binding site model consist of one bTVBT4 molecule and ten protein chains at the side where the ligand was bound (see Fig. S13). Next, for each model, a simulation box of size 15 nm x 9 nm x 9 nm was created and filled with water molecules and counter anions Cl^-. Further, after the energy minimization and equilibration process of a system, the MD production

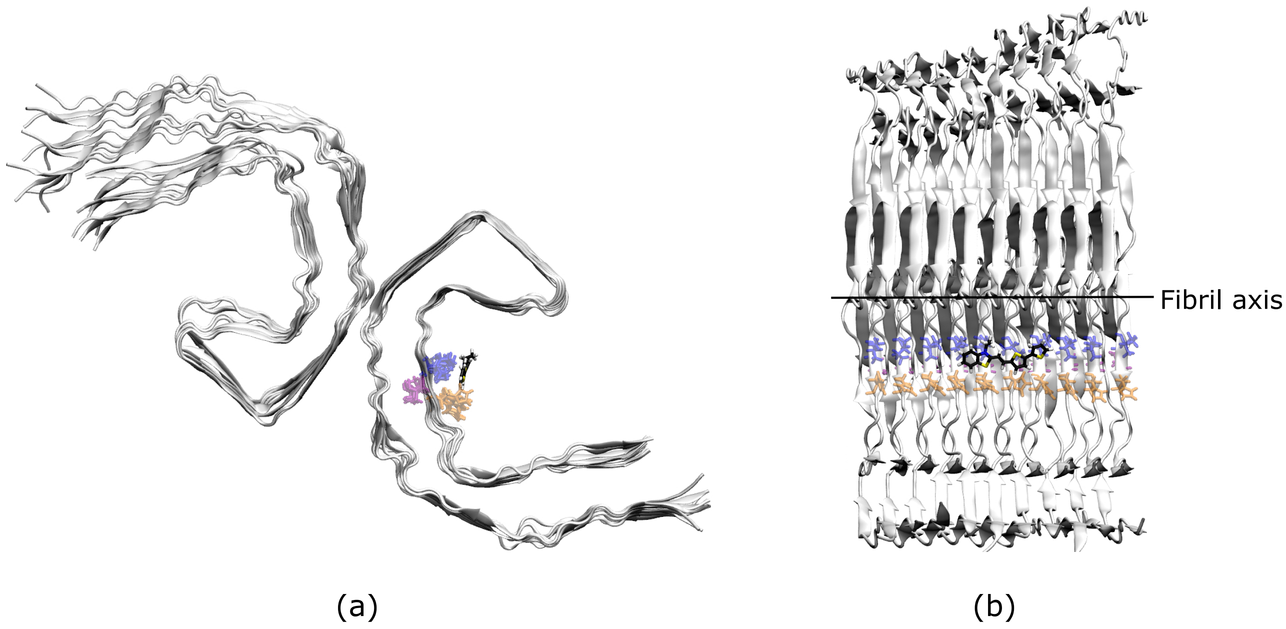

Figure S13: Side (a) and top (b) view of the small binding site tau model cropped from the full periodic tau model.

run of 250 ns in NVT was performed on two parallel trajectories for each major mode (i.e., 500 ns time for each major mode). During the whole procedure, position restraints on the outer-most tau protein chains were applied to maintain adjacent chains distance. The results obtained from this short model of binding modes are summarized in Fig. S14 and Fig. S15.

Trj 1 — DL

	1	2	3	4
1	21	5	1	0
2	7	64	0	0
3	0	0	2	5
4	0	2	4	40

Trj 1 — DR

	5	6	7	8
5	1	0	1	0
6	1	25	0	38
7	0	0	1	2
8	0	39	1	171

Trj 1 — UL

	9	10	11	12
9	0	2	0	0
10	1	40	0	3
11	1	0	48	5
12	0	2	6	63

Trj 1 — UR

	13	14	15	16
13	0	0	0	0
14	0	0	0	0
15	0	0	3	5
16	0	0	6	106

Trj 2 — DL

	1	2	3	4
1	60	1	1	0
2	1	6	0	0
3	2	0	24	1
4	0	0	2	22

Trj 2 — DR

	5	6	7	8
5	1	0	1	0
6	0	31	0	32
7	2	0	37	2
8	0	32	2	103

Trj 2 — UL

	9	10	11	12
9	3	0	0	0
10	0	0	0	1
11	1	0	37	4
12	0	1	5	115

Trj 2 — UR

	13	14	15	16
13	0	0	0	0
14	0	0	0	0
15	0	0	6	10
16	0	0	11	133

Figure S14: Time spent (ns) in each minor mode (diagonal) and number of exchanges between the minor modes (non-diagonal) from row to column. Characterized based on four major modes. Data obtained from two trajectories simulated for 250 ns for each major mode.

	Trajectories 1st time											
Initial Mode	Modes explored	Time (ns)	Coul avg	Coul std	Coul var	LJ avg	LJ std	LJ var	Tot (LJ+Coul)	Tot std	Tot var	Avg. of 4 modes
DLTO (4)	1	21	-14.3	6.1	37.6	-97.4	7.6	58.3	-111.7	9.8	95.9	-112.5 ± 10
	2	64	-13.9	6.2	38.0	-96.3	8.0	64.4	-110.2	10.1	102.4	
	3	2	-12.5	6.0	36.3	-98.5	8.3	68.8	-111.0	10.3	105.1	
	4	40	-17.4	7.2	52.3	-99.8	8.0	64.0	-117.1	10.8	116.3	
DRTI (7)	5	1	-10.7	3.9	14.9	-103.3	8.8	78.2	-114.1	9.7	93.1	-117.8 ± 11
	6	25	-14.8	6.2	38.9	-104.2	8.4	71.0	-119.0	10.5	109.8	
	7	1	-8.4	2.7	7.3	-96.1	9.1	83.1	-104.5	9.5	90.4	
	8	171	-23.5	9.8	95.8	-110.1	8.8	77.9	-133.6	13.2	173.7	
ULTO (12)	9	~0	-28.1	8.7	76.5	-81.6	8.6	73.6	-109.7	12.3	150.2	-121.3 ± 13
	10	40	-31.5	11.5	131.3	-97.0	8.0	63.7	-128.5	14.0	195.0	
	11	48	-25.5	9.6	91.9	-96.5	8.9	79.7	-121.9	13.1	171.6	
	12	63	-30.1	8.7	76.5	-95.3	10.2	104.3	-125.4	13.4	180.8	
URTO (16)	13	0										-132.0 ± 9
	14	0										
	15	3	-28.7	9.3	86.3	-102.9	8.0	64.5	-131.7	12.3	150.8	
	16	106	-28.6	9.1	83.3	-103.8	7.5	56.6	-132.4	11.8	139.9	

(a)

	Trajectories 2nd time											
Initial Mode	Modes explored	Time (ns)	Coul avg	Coul std	Coul var	LJ avg	LJ std	LJ var	Tot (LJ+Coul)	Tot std	Tot var	Avg. of 4 modes
DLTO (4)	1	60	-12.3	5.0	25.1	-97.6	7.4	55.1	-109.9	9.0	80.2	-111.9 ± 10
	2	6	-11.6	5.2	27.1	-99.3	7.6	58.4	-110.9	9.2	85.5	
	3	24	-11.6	5.0	25.1	-96.0	7.5	56.3	-107.5	9.0	81.4	
	4	22	-20.5	7.9	61.6	-98.7	7.6	57.5	-119.2	10.9	119.2	
DRTI (7)	5	1	-11.3	3.9	15.4	-96.6	8.2	67.1	-107.9	9.1	82.5	-116.8 ± 11
	6	31	-15.0	6.0	36.2	-99.3	7.6	57.7	-114.3	9.7	93.9	
	7	37	-15.3	7.5	55.6	-101.4	8.5	72.2	-116.7	11.3	127.8	
	8	103	-19.3	9.6	93.1	-109.3	9.4	89.2	-128.6	13.5	182.3	
ULTO (12)	9	3	-28.1	8.7	76.5	-81.6	8.6	73.6	-109.7	12.3	150.2	-119.2 ± 11
	10	~0	-31.5	11.5	131.3	-97.0	8.0	63.7	-128.5	14.0	195.0	
	11	37	-25.5	9.6	91.9	-96.5	8.9	79.7	-121.9	13.1	171.6	
	12	115	-30.1	8.7	76.5	-95.3	10.2	104.3	-125.4	13.4	180.8	
URTO (16)	13	0										-130.9 ± 9
	14	0										
	15	6	-27.9	8.9	78.9	-103.1	8.2	66.7	-131.0	12.1	145.6	
	16	133	-29.3	9.6	91.9	-101.5	7.7	58.7	-130.8	12.3	150.5	

(b)

Figure S15: Statistics drawn from the two parallel trajectories (a and b) for each major mode (smaller binding site model) simulated for 250 ns.

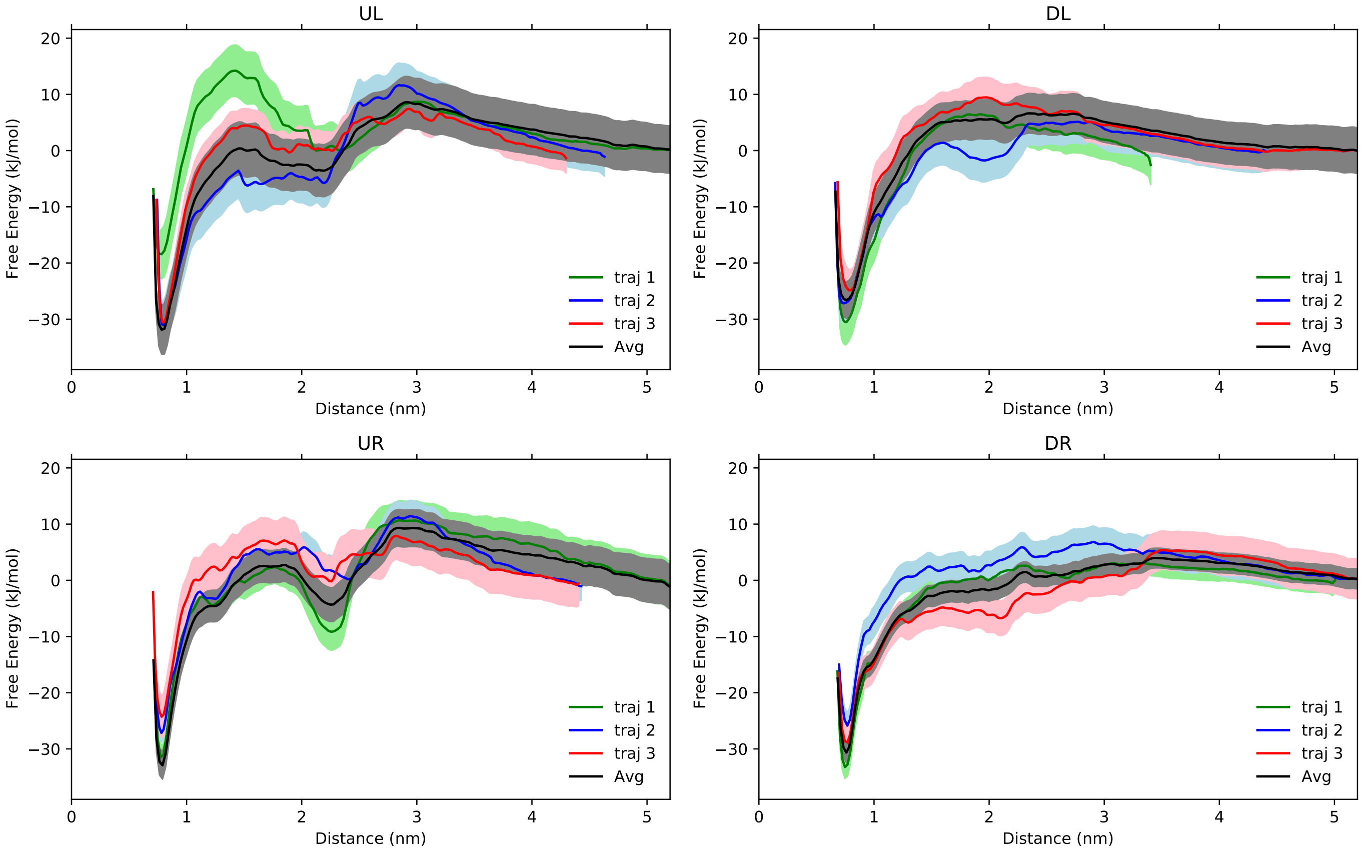

Figure S16: The PMF calculation for three pulling trajectory (green, red, blue) and its average (black) for each major modes.

1.2.6 PMF calculation details

We performed potential of mean force (PMF) calculations to obtain the height of the atomistic free-energy barrier for the bTVBT4 to bind at site A. It was achieved by using an umbrella sampling technique and a weighted histogram analysis method. Starting from the last snapshot of each major mode (short models) equilibrium MD simulation, bTVBT4 has pulled away from the binding site A to outwards. The center of mass (COM) pulling was applied between two groups Thr360 (middle residue at site A) and bTVBT4. The pulling rate of 0.001 nm/ps combined with a spring constant of 3000 kJ/mol was used. The ligand was pulled out from major modes of binding site A for about 5 nm. From these pulling trajectories, snapshots were taken to generate the starting configurations for the umbrella sampling windows. In each window, short 100 ps NVT followed by 2 ns NVT simulation was performed. Finally, analysis of results obtained from umbrella sampling was performed with the weighted histogram analysis method (WHAM).[38,39] Note that, for each major mode, three pulling simulations followed by umbrella sampling were conducted to get the average PMF curve finally (Fig. S16).

1.2.7 Absorption spectra calculation of bTVBT4 at the binding site A

To calculate absorption spectra, 300 uncorrelated snapshots were chosen based on the percentage of time spent in each minor mode and the major mode's Boltzmann percentage. Boltzmann percentage for major modes was calculated based on their respective binding energies obtained from PMF. Table S12 shows the number of snapshots chosen for each minor mode.

For each snapshot, absorption spectra were calculated by employing the polarizable embedding method. The total system was split into two regions: core and polarizable region. The core region having only a bTVBT4 molecule is treated in the quantum chemical description. The polar region consists of water molecules, counter anions (Cl^-), and amino acid residues within 20 Å of bTVBT4. For water, Ahlström charges and isotropic polarizabilities

Table S12: Number of snapshots adopted for each minor modes for the calculation of the absorption spectrum of bTVBT4 in tau environment.

Major mode	Minor mode	Number of snapshots
DL	1	3
	2	3
	3	1
	4	3
DR	5	0
	6	9
	7	6
	8	42
UL	9	1
	10	12
	11	25
	12	53
UR	13	0
	14	0
	15	5
	16	137
total		300

were used. Standard charge and isotropic polarizability for Cl^- atoms were taken from the library in the PyFrame module.[40] For the amino acid residues of tau protein, the average of charges and isotropic polarizabilities over 10 tau protofilament is used. The charges and isotropic polarizabilities were determined using the LoProp program.[41,42] The absorption calculations were carried out for the ten lowest excited states at the CAM-B3LYP(100%) and aug-cc-pVDZ basis set using the Dalton program. Subsequently, for each snapshot, the calculated absorption spectrum was Gaussian convoluted with standard deviation of 0.15 eV. The final spectral profile was obtained by taking an average over all the snapshots under consideration.

1.2.8 Planarity calculation

The planarity parameter used for the assessment of the molecular structure of bTVBT4 is adopted from Ref. 43 and reads

$$P = \sum_{i=1}^{N} \frac{||\theta_i| - 90|}{90}, \quad (2)$$

where θ_i are the dihedral angles in the molecule, and N is the number of dihedral angles for which the planarity is calculated. For the bTVBT4 case, the planarity was calculated over four dihedrals (marked in Fig. S1 (a)). Fig. S17 shows the correlation between planarity and transition energy of the first excited state, calculated in water (using 200 snapshots) and tau (using 300 snapshots) environment. The average value of planarity for bTVBT4 in water is 3.43 ± 0.20 and in tau is 3.49 ± 0.18.

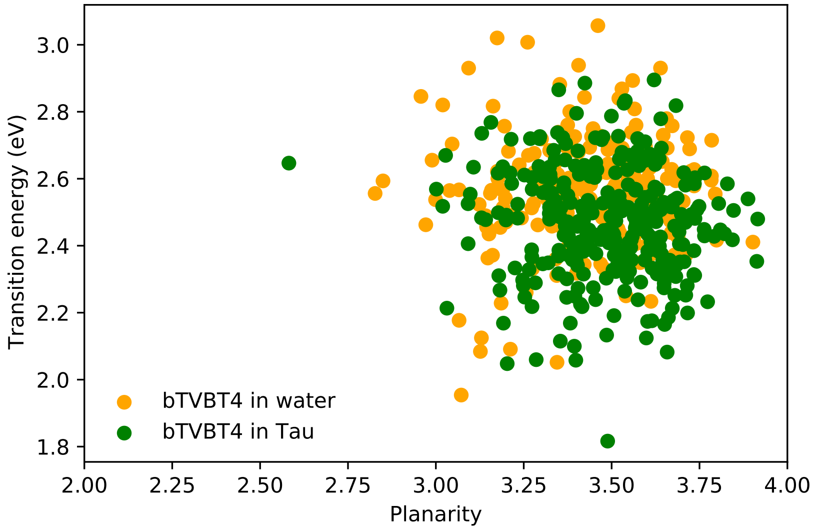

Figure S17: Correlation between planarity (defined in Eq. 2) vs. transition energy for the first excited state of bTVBT4. In total, 200 points are shown for bTVBT4 in water (orange), and 300 points shown for bTVBT4 in the tau fibril environment of binding site A (green) , corresponding to the snapshots used in spectrum calculation. The average value of planarity for bTVBT4 in water is 3.43 ± 0.20 and in tau is 3.49 ± 0.18.

1.2.9 Qualitative picture of interactions between bTVBT4 and siteA

The intramolecular and intermolecular interactions are accounted within the Columbic term (electrostatic interactions) and Lennard-Jones function (van der Waals interactions) in the forcefield. Fig. S18 shows RESP charges mapped on site A and bTVBT4 atoms. The strongest Columbic interaction is observed between the imidazole ring of His362 (having negative charge cloud) and the bTVBT4.

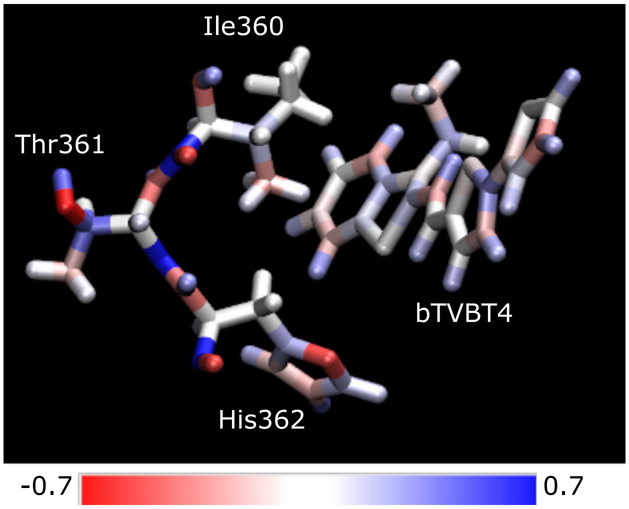

Figure S18: RESP charges used in MD simulation for site A and bTVBT4 are mapped on atoms with color code.

2 Experimental materials, methods, and supplementary results

2.1 Antibody and ligand double labelling

Frozen human brain tissue sections (10 μm, frontal cortex) with Alzheimers disease (AD) or Picks disease (PiD) pathology were fixed in 70% EtOH for 3 min at 4°C and then incubated in dH2O for 2×2 min and phosphate buffered saline (PBS, 10 mM phosphate, 140 mM NaCl, 2.7 mM KCl, pH 7.4) for 10 min at RT. Next, a permeabilization and blocking step in PBS with 0.1% triton x-100 (PBS-T) and 5% normal goat serum was performed for 1 h at RT.

Monoclonal anti-phospho-tau-antibody AT8 (Thermo Scientific) was diluted 1:500 in PBS-T with 5% normal goat serum and added to the sections. After incubation over night at 4°C, the sections were washed in PBS-T for 3×10 min and then incubated for 1 h at RT with goat anti-mouse secondary antibody conjugated to Alexa 488 (Thermo Scientific) diluted 1:400 in PBS-T with 5% normal goat serum. The sections were washed in PBS for 3x10 min and ligand bTVBT4, diluted to 100 nM in PBS, was added. After 30 min at RT, excess ligand was removed by repeated washings in PBS and the sections were mounted using Dako mounting medium for fluorescence (Agilent). The result was analyzed using an inverted Zeiss LSM 780 laser scanning confocal microscope (Zeiss) exciting the samples at 405 nm (lipofuscin), 490 nm (Alexa 488) and 580 nm (bTVBT4).

2.2 Hyperspectral and fluorescence lifetime imaging

Frozen brain tissue section (10 μm, frontal cortex) with AD or PiD pathology was fixed in 99.7% EtOH for 10 min at RT, rehydrated in 50% EtOH for 2 min, dH2O for 2×2 min and then PBS for 10 min. The section was incubated in 100 nM b-TVBT4 ligand for 30 min at RT and then washed repeatedly with PBS. After mounting with Dako mounting medium for fluorescence (Agilent), fluorescence lifetime imaging was performed using an inverted Zeiss LSM 780 laser scanning confocal microscope (Zeiss) equipped with a 32 channel QUASAR GaAsP spectral array detector. Emitted photons were routed through the direct coupling confocal port of the Zeiss LSM 780 scanning unit and detected by a Becker & Hickl HPM-100-40 hybrid photomultiplier tube (Becker & Hickl GmbH). Data were recorded by a Simple-Tau 152 system (SPC-150 TCSPC FLIM module) with the instrument recording software SPCM version 9.42 in the FIFO image mode using 256 time-channels. A Plan-Apochromat 20×/1.3 Oil DIC objective lens was used, and the pinhole set to 20.2 μm. For excitation at 535 nm, a pulsed tunable In Tune laser with a repetition rate of 40 MHz was used. Data were analyzed using SPCImage version 3.9.4. Emission spectra from bTVBT4 bound to tau pathology in AD were recorded between 561 nm to 687 nm using an inverted LSM

780 confocal microscope (Carl Zeiss, Oberkochen, Germany) and excitation spectra were collected with the same microscope system using a tunable In Tune laser and the excitation wavelength was scanned between 490 to 600 nm having the emission fixed at 612 nm.

When bound to tau aggregates in AD, bTVBT4 displayed strikingly longer decay times, 1.7 to 2.4 ns, than for the ligand in different solvents, verifying that bTVBT4 adopts a distinct conformation when bound to the aggregates.

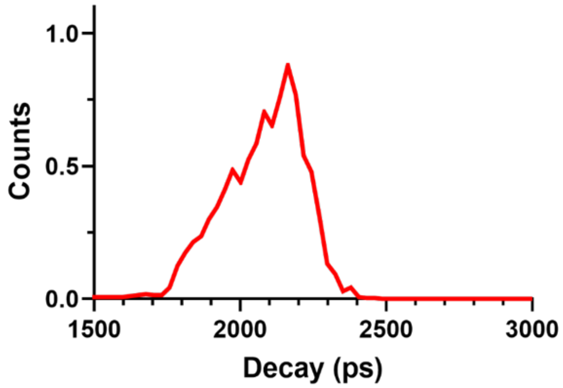

Figure S19: Intensity-weighted mean lifetime distributions of bTVBT4-stained tau deposits in AD brain tissue sections. The fluorescence lifetimes were collected with excitation at 535 nm.

2.3 Spectroscopy

Steady-state absorption spectra were recorded using a Shimadzu UV-1601PC spectrophotometer. Measurements were performed with 10 mm quartz cuvettes (Hellma Precision). Steady-state photoluminescence measurements were carried out employing a PTI Quantamaster 8075-22 (Horiba Scientific) equipped with Double Monochromator 300 spectrometer chambers for both excitation and emission. A Hamamatsu R928 PMT was used for detection in the range 185–950 nm. A OB-75X (75 W Xenon arc lamp) was used as the light source. Data acquisition and basic data-handling of steady state luminescence data were carried out with the Felix Data Analysis software and further processed and presented using Origin Pro.

Time-correlated single photon counting (TC-SPC) was used to register and analyze decay

traces of bTVBT4 in a selection of solvents using an IBH system as described recently in Ref. 44. In the experiments a picosecond laser diode at 469 nm was used for excitation and the emission was recorded at 600 nm with a 16 nm slit.

Table S13: Experimental absorption (λ_{abs} and ΔE) and emission (λ_{em}) maxima and quantum efficiencies for bTVBT4 in different solvents with dielectric constants ε_r.[a]

Solvent	λ_{abs} (nm)	ΔE (eV)	λ_{em} (nm)	QE (%)	ε_r
PBS (H_2O)	463	2.678	604	0.24±0.03	81
methanol	476	2.605	605	0.22±0.02	33
ethanol	479	2.588	604	0.29±0.03	24
n-butanol	490	2.530	605	0.39±0.04	18
n-pentanol	492	2.520	604	0.76±0.09	15
n-octanol	495	2.505	603	0.67±0.08	10
acetonitrile	471	2.632	607	0.23±0.03	37
DMF	477	2.599	614	0.74±0.09	37
$CHCl_3$	509	2.436	605	0.05±0.007	4.8

[a]Dielectric constants are taken from `http://www.stenutz.eu/chem/solv6.php?name=octanol` (n-octanol) and `https://www.engineeringtoolbox.com/liquid-dielectric-constants-d_1263.html` (others).

The blue shift of the absorption spectra of bTVBT4 in more polar solvent might be due to a negative solvatochromic effect associated with an excited state that is less polar than the ground state. In such a situation the ground state becomes more effectively relaxed in the reaction field of the solvent and the ground-to-excited state energy difference thus becomes larger upon introducing more polar solvent. Interestingly, there is almost no, or at least very small, differences in the emission spectra with respect to solvent polarity, corroborating the notion that the excited state is less polar.

Attempted lifetime measurements of bTVBT4 at a concentration of 2.5 μM in selected solvents showed very rapid decays, at the limit of the resolution of our TC-SPC system. Typically, for polar protic solvents such as PBS, methanol, and ethanol, the lifetimes were in the order of 10–20 ps (±10 ps) and for non-protic solvents such as acetonitrile, $CHCl_3$, and DMF, around 30 ps (±5 ps). Two representative decay traces are shown in Fig. S21.

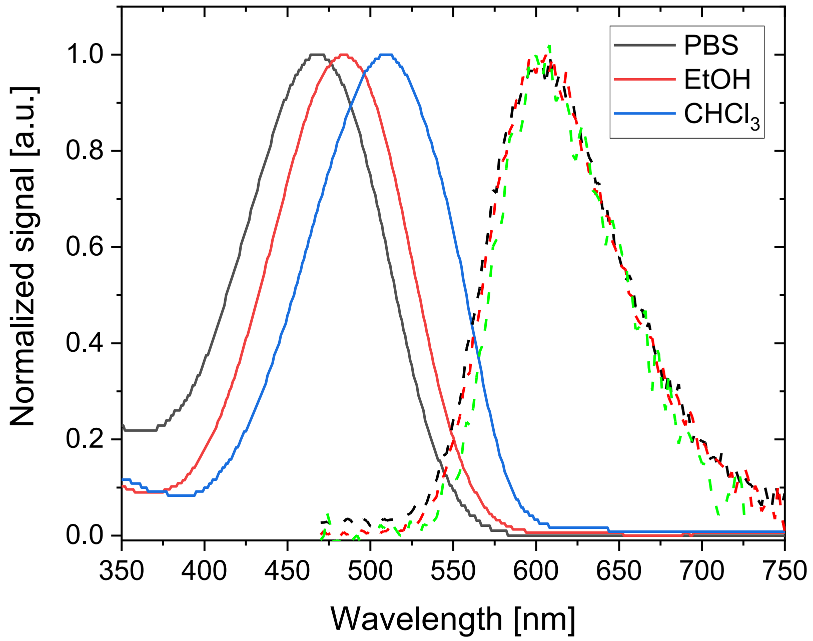

Figure S20: Representative absorption (solid) and emission (dashed) spectra of bTVBT4 in various solvents: PBS, ethanol and chloroform. All spectra are normalized. Photophysical parameters are summarized in Table S13.

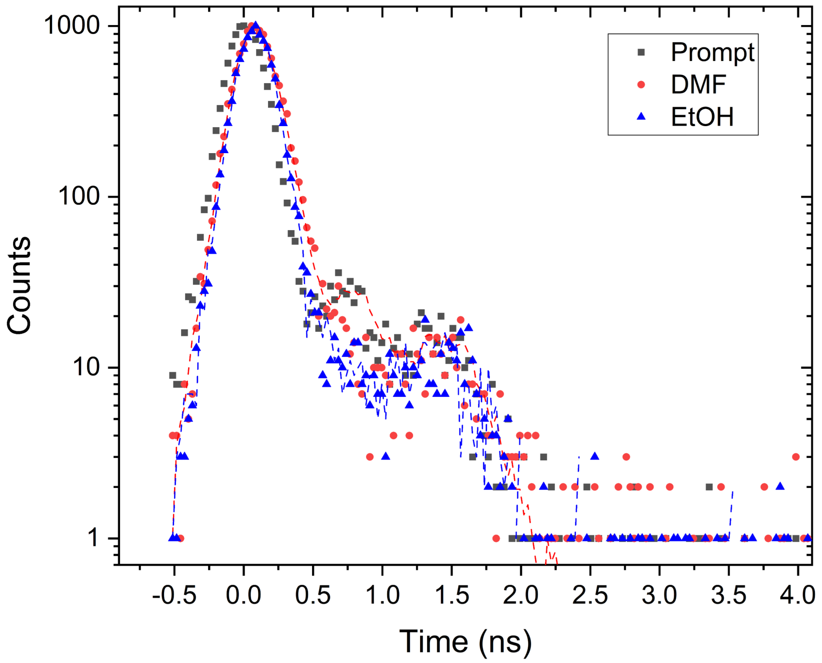

Figure S21: Representative TC-SPC decays of bTVBT4 in EtOH and DMF (2.5 mM) along with attempted re-convolution fits (dashed lines). "Prompt" denotes the system response acquired from the scatter of the pure solvent at the excitation wavelength.

References

(1) Frisch, M. J.; Trucks, G. W.; Schlegel, H. B.; Scuseria, G. E.; Robb, M. A.; Cheeseman, J. R.; Scalmani, G.; Barone, V.; Petersson, G. A.; Nakatsuji, H. et al. Gaussian16 Revision B.01. 2016; Gaussian Inc. Wallingford CT.

(2) Aidas, K.; Angeli, C.; Bak, K. L.; Bakken, V.; Bast, R.; Boman, L.; Christiansen, O.; Cimiraglia, R.; Coriani, S.; Dahle, P. et al. The Dalton Quantum Chemistry Program system. *Wiley Interdiscip. Rev. Comput. Mol. Sci.* **2014**, *4*, 269–284.

(3) Becke, A. D. Density-Functional Thermochemistry. III. The Role of Exact Exchange. *J. Chem. Phys.* **1993**, *98*, 5648.

(4) Lee, C.; Yang, W.; Parr, R. G. Development of the Colle-Salvetti correlation-energy formula into a functional of the electron density. *Phys. Rev. B* **1988**, *37*, 785–789.

(5) Stephens, P. J.; Devlin, F. J.; Chabalowski, C. F.; Frisch, M. J. Ab Initio Calculation of Vibrational Absorption and Circular Dichroism Spectra Using Density Functional Force Fields. *The Journal of Physical Chemistry* **1994**, *98*, 11623–11627.

(6) Petersson, G. A.; Bennett, A.; Tensfeldt, T. G.; AlLaham, M. A.; Shirley, W. A.; Mantzaris, J. A complete basis set model chemistry. I. The total energies of closedshell atoms and hydrides of the firstrow elements. *The Journal of Chemical Physics* **1988**, *89*, 2193–2218.

(7) Petersson, G. A.; AlLaham, M. A. A complete basis set model chemistry. II. Openshell systems and the total energies of the firstrow atoms. *The Journal of Chemical Physics* **1991**, *94*, 6081–6090.

(8) Yanai, T.; Tew, D. P.; Handy, N. C. A new hybrid exchange–correlation functional using the Coulomb-attenuating method (CAM-B3LYP). *Chem. Phys. Lett* **2004**, *393*, 51–57.

(9) Dunning, T. H. Gaussian Basis Sets for use in Correlated Molecular Calculations. I. The Atoms Boron Through Neon and Hydrogen. *J. Chem. Phys.* **1989**, *90*, 1007–1023.

(10) Kendall, R. A.; Dunning Jr, T. H.; Harrison, R. J. Electron affinities of the first-row atoms revisited. Systematic basis sets and wave functions. *The Journal of chemical physics* **1992**, *96*, 6796–6806.

(11) Champion, P.; Albrecht, A. On band shapes of electronic transitions in the multimode weak coupling limit. *J. Chem. Phys.* **1980**, *72*, 6498–6506.

(12) Myers, A. B.; Mathies, R. A. Resonance Raman intensities: a probe of excited-state structure and dynamics. *Biological applications of Raman spectroscopy* **1987**, *2*, 1–58.

(13) Christiansen, O.; Artiukhin, D.; Godtliebsen, I.; Gras, E.; Győrffy, W.; Hansen, M.; Hansen, M.; Kongsted, J.; Klinting, E.; König, C. et al. MidasCpp (molecular interactions, dynamics and simulation chemistry program package in C++). 2021; `https://midascpp.gitlab.io/`.

(14) Moitra, T.; Madsen, D.; Christiansen, O.; Coriani, S. Vibrationally resolved coupled-cluster x-ray absorption spectra from vibrational configuration interaction anharmonic calculations. *J. Chem. Phys.* **2020**, *153*, 234111.

(15) Madsen, D.; Christiansen, O.; Norman, P.; König, C. Vibrationally resolved emission spectra of luminescent conjugated oligothiophenes from anharmonic calculations. *Phys. Chem. Chem. Phys.* **2019**, *21*, 17410–17422.

(16) Sparta, M.; Toffoli, D.; Christiansen, O. An adaptive density-guided approach for the generation of potential energy surfaces of polyatomic molecules. *Theor. Chem. Acc.* **2009**, *123*, 413–429.

(17) Toffoli, D.; Sparta, M.; Christiansen, O. Accurate multimode vibrational calculations

using a B-spline basis: theory, tests and application to dioxirane and diazirinone. *Mol. Phys.* **2011**, *109*, 673–685.

(18) Wang, J.; Wang, W.; Kollman, P. A.; Case, D. A. Automatic atom type and bond type perception in molecular mechanical calculations. *Journal of Molecular Graphics and Modelling* **2006**, *25*, 247–260.

(19) Wang, J.; Wolf, R. M.; Caldwell, J. W.; Kollman, P. A.; Case, D. A. Development and testing of a general amber force field. *Journal of Computational Chemistry* **2004**, *25*, 1157–1174.

(20) Lelanda, B.; Paul, D.; Krueger, B.; Walker, R. `http://ambermd.org/tutorials/advanced/tutorial1/section1.htm`.

(21) Bayly, C. I.; Cieplak, P.; Cornell, W.; Kollman, P. A. A well-behaved electrostatic potential based method using charge restraints for deriving atomic charges: the RESP model. *The Journal of Physical Chemistry* **1993**, *97*, 10269–10280.

(22) Berendsen, H.; van der Spoel, D.; van Drunen, R. GROMACS: A message-passing parallel molecular dynamics implementation. *Computer Physics Communications* **1995**, *91*, 43–56.

(23) Lindahl, E.; Hess, B.; van der Spoel, D. GROMACS 3.0: a package for molecular simulation and trajectory analysis. *Molecular modeling annual* **2001**, *7*, 306–317.

(24) Van Der Spoel, D.; Lindahl, E.; Hess, B.; Groenhof, G.; Mark, A. E.; Berendsen, H. J. C. GROMACS: Fast, flexible, and free. *Journal of Computational Chemistry* **2005**, *26*, 1701–1718.

(25) Hess, B.; Kutzner, C.; van der Spoel, D.; Lindahl, E. GROMACS 4: Algorithms for Highly Efficient, Load-Balanced, and Scalable Molecular Simulation. *Journal of Chemical Theory and Computation* **2008**, *4*, 435–447.

(26) Sousa da Silva, A. W.; Vranken, W. F. ACPYPE - AnteChamber PYthon Parser interfacE. *BMC Research Notes* **2012**, *5*, 367.

(27) Wildman, J.; Repiščák, P.; Paterson, M. J.; Galbraith, I. General Force-Field Parametrization Scheme for Molecular Dynamics Simulations of Conjugated Materials in Solution. *Journal of Chemical Theory and Computation* **2016**, *12*, 3813–3824.

(28) Virtanen, P.; Gommers, R.; Oliphant, T. E.; Haberland, M.; Reddy, T.; Cournapeau, D.; Burovski, E.; Peterson, P.; Weckesser, W.; Bright, J. et al. SciPy 1.0: Fundamental Algorithms for Scientific Computing in Python. *Nature Methods* **2020**, *17*, 261–272.

(29) Diaz-Quijada, G. A.; Weinberg, N.; Holdcroft, S.; Pinto, B. M. Investigation of Barriers To Conformational Interchange in Oligothiophenes and Oligo(Thienyl)furans. *The Journal of Physical Chemistry A* **2002**, *106*, 1266–1276.

(30) Maier, J. A.; Martinez, C.; Kasavajhala, K.; Wickstrom, L.; Hauser, K. E.; Simmerling, C. ff14SB: Improving the Accuracy of Protein Side Chain and Backbone Parameters from ff99SB. *Journal of Chemical Theory and Computation* **2015**, *11*, 3696–3713.

(31) Jorgensen, W. L.; Chandrasekhar, J.; Madura, J. D.; Impey, R. W.; Klein, M. L. Comparison of Simple Potential Functions for Simulating Liquid Water. *J. Chem. Phys.* **1983**, *79*, 926–935.

(32) Darden, T.; York, D.; Pedersen, L. Particle mesh Ewald: An N log(N) method for Ewald sums in large systems. *The Journal of Chemical Physics* **1993**, *98*, 10089–10092.

(33) Hess, B.; Bekker, H.; Berendsen, H. J. C.; Fraaije, J. G. E. M. LINCS: A linear constraint solver for molecular simulations. *Journal of Computational Chemistry* **1997**, *18*, 1463–1472.

(34) Olsen, J. M. H.; Kongsted, J. *Advances in Quantum Chemistry, Chapter 3 – Molecular Properties Through Polarizable Embedding*; Academic Press: San Diego, USA, 2011; Vol. 61.

(35) List, N. H.; Norman, P.; Kongsted, J.; Jensen, H. J. Aa. A Quantum-Mechanical Perspective on Linear Response Theory Within Polarizable Embedding. *J. Chem. Phys.* **2017**, *146*, 234101.

(36) Ahlström, P.; Wallqvist, A.; Engström, S.; Jönsson, B. A Molecular Dynamics Study of Polarizable Water. *Mol. Phys.* **1989**, *68*, 563–581.

(37) Fitzpatrick, A. W. P.; Falcon, B.; He, S.; Murzin, A. G.; Murshudov, G.; Garringer, H. J.; Crowther, R. A.; Ghetti, B.; Goedert, M.; Scheres, S. H. W. Cryo-EM structures of tau filaments from Alzheimers disease. *Nature* **2017**, *547*, 185–190.

(38) Kumar, S.; Rosenberg, J. M.; Bouzida, D.; Swendsen, R. H.; Kollman, P. A. THE weighted histogram analysis method for free-energy calculations on biomolecules. I. The method. *Journal of Computational Chemistry* **1992**, *13*, 1011–1021.

(39) Hub, J. S.; de Groot, B. L.; van der Spoel, D. g_whamA Free Weighted Histogram Analysis Implementation Including Robust Error and Autocorrelation Estimates. *Journal of Chemical Theory and Computation* **2010**, *6*, 3713–3720.

(40) Olsen, J. M. H. {PyFraME}: Python Framework for Fragment-based Multiscale Embedding (version 0.2.0). 2018; `https://doi.org/10.5281/zenodo.1443314`.

(41) Gagliardi, L.; Lindh, R.; Karlström, G. Local Properties of Quantum Chemical Systems: The LoProp Approach. *J. Chem. Phys.* **2004**, *121*, 4494–4500.

(42) Vahtras, O. LoProp for Dalton. 2014; `http://dx.doi.org/10.5281/zenodo.13276`.

(43) Sjöqvist, J.; Maria, J.; Simon, R. A.; Linares, M.; Norman, P.; Nilsson, K. P. R.;

Lindgren, M. Toward a molecular understanding of the detection of amyloid proteins with flexible conjugated oligothiophenes. *J. Phys. Chem. A* **2014**, *118*, 9820–9827.

(44) Gustafsson, C.; Shirani, H.; Leira, P.; Rehn, D. R.; Linares, M.; Nilsson, K. P. R.; Norman, P.; Lindgren, M. Deciphering the Electronic Transitions of Thiophene-Based Donor-Acceptor-Donor Pentameric Ligands Utilized for Multimodal Fluorescence Microscopy of Protein Aggregates. *ChemPhysChem* **2021**, *22*, 323–335.
